# Review of Brazilian jawfishes of the genus *Opistognathus* with descriptions of two new species (Teleostei, Opistognathidae)

**DOI:** 10.3897/zookeys.794.26789

**Published:** 2018-11-01

**Authors:** William F. Smith-Vaniz, Luke Tornabene, Raphael M. Macieira

**Affiliations:** 1 Florida Museum of Natural History, University of Florida, Gainesville, FL 32611-7800, USA University of Florida Gainesville United States of America; 2 School of Aquatic and Fishery Sciences and Burke Museum of Natural History and Culture, University of Washington, 1122 NE Boat Street, Seattle, WA 981045, USA University of Washington Seattle United States of America; 3 Laboratório de Ecologia Marinha, Complexo Biopráticas, Universidade Vila Velha, Vila Velha, ES, 29102-770, Brazil Universidade Vila Velha Vila Velha Brazil; 4 Laboratório de Ictiologia, Departamento de Oceanografia e Ecologia, Universidade Federal do Espírito Santo, Vitória, ES, 29075-910, Brazil Universidade Federal do Espírito Santo Vitória Brazil

**Keywords:** Brazilian Province, Fernando de Noronha Archipelago, reef fish, Trindade-Martin Vaz insular complex, Vitória-Trindade Seamount Chain.

## Abstract

A new species of jawfish, *Opistognathusthionyi***sp. n.**, is described from the Vitória-Trindade Chain and Fernando de Noronha Archipelago off Brazil, a disjunct distribution of ca. 1,800 km. *Opistognathusthionyi* and its allopatric Caribbean sister-species, *Opistognathusmaxillosus*, both have a wide, fan-like upper margin of the subopercular flap and mostly over-lapping meristic data. The new species differs from *O.maxillosus* in having the darkest spot on the spinous dorsal fin, when present, between spines 2–5, versus always present between spines 6–9, the buccal area surrounding the esophageal opening pale versus very dark and fewer oblique scale rows in longitudinal series (45–52 vs. 69–85). A second new species, *Opistognathusvicinus***sp. n.**, known from Brazil’s mainland, has completely over-lapping meristic values with its allopatric Caribbean sister-species *O.whitehursti*, but differs in lacking vomerine teeth and a supramaxilla and retaining the juvenile color pattern of the latter species in adults. Diagnoses, photographs, an identification key, and distributional maps are given for all Brazilian species of *Opistognathus*.

Molecular phylogenetic analysis of partial cytochrome c oxidase subunit-I sequences indicates that specimens of the two allopatric pairs, *O.thionyi* – *O.maxillosus* and *O.vicinus* – *O.whitehursti*, form reciprocally monophyletic groups that differ from each other on average by 9 to 11%, with less than 1% average pair-wise genetic distance within-species. Similar patterns of phylogenetic structure were observed between reciprocally monophyletic (predominately allopatric) groups within nominal species of *Opistognathusaurifrons*, suggesting the possibility of at least two additional undescribed species from the Brazilian Province.

## Introduction

Members of the family Opistognathidae range in size from 2 to 40 cm standard length and occur in all tropical oceans except the eastern Atlantic. Their natural history is of special interest because they construct burrows on sandy or rubble bottoms, near reefs using small stones or coral fragments to maintain structural integrity, and the males orally brood the egg clutches (Hess 1993), which are occasionally aerated and cleaned. They are strongly territorial, and a single fish occupies each burrow. Jawfishes of the genus *Opistognathus* Cuvier, 1816 are widely distributed in tropical waters of the western Atlantic Ocean, where 14 valid species occur in depths of 0.3 to at least 375 m (Smith-Vaniz 1997; Smith-Vaniz 2017). An additional eleven species occur in the eastern Pacific (Bussing and Lavenberg 2003) but no species are shared by both oceans.

Prior to this study, four species of *Opistognathus* were known from Brazil. Two species, *O.cuvierii* Valenciennes, 1836 and *O.brasiliensis* Smith-Vaniz, 1997, are endemic to Brazil, and one species, *O.lonchurus* Jordan & Gilbert, 1882, has both Brazilian and broad Caribbean distributions. The fourth Brazilian species is Opistognathusaff.aurifrons, and it includes two genetically-distinct allopatric Brazilian morphotypes. The only other jawfish known from Brazil is *Lonchopisthuslemur* (Myers, 1935), a widely distributed deep-water species (Smith-Vaniz and Walsh 2017). Here, we (1) describe two new species of *Opistognathus* from Brazil, *O.thionyi* sp. n., and *O.vicinus* sp. n., and compare them with their Caribbean sister species, bringing the total number of species in Brazil to six, (2) present abbreviated descriptions, photographs or illustrations and distribution maps of all the other species of *Opistognathus* known from the Brazilian Province, (3) provide a partial molecular phylogeny based on sequence data from mitochondrial cytochrome oxidase I for available material of Atlantic species of *Opistognathus*, and (4) compare and discuss O.aff.aurifrons Brazilian populations. For the latter populations, we do not assign new scientific names to them because additional analysis of their complex phylogenetic relationships with Caribbean populations of the *O.aurifrons* complex is required to confidently determine their taxonomic status. Comparison of the six species of *Opistognathus* known from the Brazilian Province is given in Table 1.

**Table 1.** Summary of selected characters in Brazilian species of *Opistognathus*. Exceptional values in parentheses.

Characters	* O. thionyi *	* O. vicinus *	* O. brasiliensis *	* O. cuvierii *	* O. lonchurus ^1^*	* O. aff. aurifrons *
Dorsal fin	XI, 16 (15)	XI, 14	XI, 16	XI, 16	XI, 12 (13)	XI, 14–15
Anal fin	III, 15 (14)	II–III, 13 (12)	III, 15–16	III, 16	III, 12 (13)	III, 14–15
Caudal vertebrae	18	17	18	19	16	17
Supraneurals	0	0	2	1–2	1	0
Body scale rows	45–52	43–47	59–75	60–72	63–76	66–76
LL terminus	3–5	1–3	3–4	2–3	2–4	6–9
Unbranched dorsal rays	0	0–1	1	1	5–8	6–11
Vomerine teeth	1	0	1–3	2	2–7	1–3
Nasal cirrus	yes	yes	yes	yes	no	no
Upper jaw fimbriate	no	no	yes	yes	no	no
Supramaxilla present	no	no	yes	yes	yes	yes
Subopercle flap wide	yes	no	no	no	no	no
Dorsal-fin spines stiff with fleshy tabs on tips^2^	yes	yes	no	no	no	no
Buccal pigmentation	no	no	yes	yes	no	no
Caudal fin banded	yes	yes	no	yes	no	no
Spinous dorsal-fin with dark spot or ocellus	yes or no	yes	yes	yes	no	no

^1^ Includes data from non-Brazilian specimens. ^2^ Fleshy spine tabs present only in adults.

## Materials and methods

Institutional abbreviations mostly follow Fricke and Eschmeyer (2018) and include the following collection depositories:

**ANSP**Academy of Natural Sciences of Drexel University, Philadelphia, Pennsylvania

**CAS**California Academy of Sciences, San Francisco, California

**CIUFES** Coleção Ictiológica, Departamento de Oceanografia e Ecologia, Universidade Federal do Espírito, Vitória, Espírito Santo, Brazil

**FMNH** Division of Fishes, Department of Zoology, Field Museum of Natural History, Chicago, Illinois

**FSBC** Florida Fish & Wildlife Conservation Commission, Fish & Wildlife Research Institute, St. Petersburg, Florida

**MCZ**Museum of Comparative Zoology, Harvard University, Ichthyology Department, Cambridge, Massachusetts

**MNHN**Museum National d’Histoire Naturelle, Systématique et Évolution, Laboratoire d’Ichyologie Générale et Appliquée, Paris

**MNRJ** Museu Nacional, Departmento de Vertebrados, Setor de Ictiologia, Universidade Federal do Rio de Janeiro, Brazil

**MZUSP**Universidade de São Paulo, Museu de Zoologia, São Paulo, Brazil

**NPM** Núcleo em Ecologia e Desenvolvimento Socio-Ambiental (NUPEM), Universidade Federal do Rio de Janeiro (UFRJ), Macaé, Brazil

**SIO**Scripps Institution of Oceanography, Marine Vertebrate Collection, La Jolla, California

**SU** Stanford University, collection transferred to CAS

**UF**University of Florida, Museum of Natural History, Gainesville, Florida

**UFPB**Departamento de Sistemática e Ecologia, Universidade Federal da Paraíba, João Pessoa, Paraíba, Brazil

**USNM**Smithsonian Institution National Museum of Natural History, Department of Vertebrate Zoology, Division of Fishes, Washington, D.C.

**ZUEC**Universidade Estadual de Campinas, Museu de História Natural “Prof. Dr. Adão José Cardoso”, Museu de Zoologia, São Paulo, Brazil

Median fin-ray counts, and characters associated with the vertebral column were usually taken from radiographs. The last two elements in the dorsal and anal fins have their bases in close approximation (“split to base” condition) and were counted as one ray in accord with the general practice of most authors, although the ultimate element has a separate rudimentary pterygiophore or stay. Pectoral-fin ray counts are reported for one side only and include the uppermost rudimentary ray. Caudal-fin ray counts separated by a plus indicate rays associated with the dorsal and then the ventral hypural plate. Vertebral counts are presented as a formula: precaudal + caudal. The lateral-line terminus refers to the base of the posteriomost segmented dorsal-fin ray below which the lateral line ends. The number of oblique body scale rows is only an approximation due to the irregular size and arrangement of individual scale rows. Included in this count are all anteroventrally aligned scale rows in a longitudinal series from above the tip of the opercular flap to the base of the caudal fin (counts of posteroventrally aligned scale rows will result in lower values). The gill raker at the junction of the upper and lower limbs of the first gill arch is included in the lower-limb count; care was taken not to overlook rakers (often very small) at the ends of the gill arch. Counts of gill rakers were usually made only on the right side of specimens. English common names of species, if available, are those of Page et al. (2013).

Specimen sizes in material examined are given as mm SL (standard length) rounded to the nearest 0.1 mm, with number of specimens and size range given in parentheses. Measurements of paratypes indicated by an asterisk were compared with those of the holotypes. Cleared and stained specimens are indicated as “C&S”. All measurements were made with needle-point digital calipers and recorded to the nearest 0.1 mm. Measurements of paratypes indicated by an asterisk were compared with holotypes of the new species. Head length is the distance from the middle of the upper lip to the posterodorsal tip of the opercular flap. Postorbital-jaw length is a straight-line measurement from the posterior orbital margin at its junction with the rigid sphenotic bone to a vertical from the posterior end of the upper jaw. Postorbital-jaw ratio is the postorbital jaw length divided by the orbit diameter. Orbit diameter is a diagonal (posterodorsal to anteroventral) measurement of the bony orbit; the posterodorsal point of origin is the rigid sphenotic margin. Body depth is a vertical measurement from the origin of the anal fin. Caudal-peduncle depth is a vertical measurement from the narrowest part of the caudal peduncle. In the color pattern descriptions, stripes refer to markings aligned with the longitudinal axis of the body and bands or bars refer to markings aligned with the vertical axis of the body.

We sequenced a segment of the mitochondrial gene cytochrome c oxidase subunit-I (COI) for 22 samples of *Opistognathus*, including one specimen of *O.whitehursti* from St. Croix and three of *O.vicinus* from Brazil, eight specimens of *O.thionyi* and eight specimens of O.aff.aurifrons, and one specimen each of *O.lonchurus* and *O.brasiliensis*. Whole genomic DNA was extracted using a Qiagen DNEasy Blood and Tissue kit per manufacturers’ protocols. Primers for PCR and sequencing reactions were COH6 and COL6 (Shubart et al. 2007) or GobyL6468 and GobyH7696 (Thacker 2003). Sequences were aligned with several other opistognathid sequences from GenBank and BOLD (Barcode of Life Database, www.boldsystems.org), plus a sequence from *Lonchopisthusmicrognathus* (Poey, 1860) as an outgroup, for a total of 103 sequences. Sequences were assembled and aligned in *Geneious* v.10.0.9. The best fitting substitution model was chosen using PartitionFinder (Lanfear et al. 2016). Phylogenetic analyses were conducted using maximum likelihood and Bayesian methods in RAxML version 8 (Stamatakis 2014) and MrBayes ver. 3.2.6 (Ronquist et al. 2012), respectively. A distance matrix was created using mean between-group pairwise genetic distances (Table 2); and a haplotype network was created using Haploviewer (Salzburger et al. 2011). GenBank or BOLD accession numbers are listed in the Appendix.

**Table 2.** Mean between-group p-distances. Shaded values on the diagonal are mean within-group p-distances for groups with more than one sequence. The number of base differences per site from averaging over all sequence pairs between groups are shown. The analysis involved 103 nucleotide sequences. Codon positions included 1st+2nd+3rd+noncoding. All positions containing gaps and missing data were eliminated. There are a total of 103 positions in the final dataset. Evolutionary analyses were conducted in MEGA7 [1]. Caribbean Clade 1 = Florida, Bahamas, Caribbean; Caribbean Clade 2 = Aruba and Curacao

Species	* L. micrognathus *	* O. robinsi *	* O. macrognathus *	* O. brasiliensis *	* O. thionyi *	* O. maxillosus *	* O. whitehursti *	* O. vicinus *	* O. lonchurus *	*O.aurifrons* – Caribbean Clade 1	*O.aurifrons* – Caribbean Clade 2	*O.aurifrons* – Fernando de Noronha	*O.aurifrons* – Brazil mainland
* Lonchopisthus micrognathus *	n/a												
* Opistognathus robinsi *	0.195	n/a											
* Opistognathus macrognathus *	0.191	0.161	0.003										
* Opistognathus brasiliensis *	0.181	0.141	0.120	n/a									
* Opistognathus thionyi *	0.176	0.135	0.138	0.132	0.002								
* Opistognathus maxillosus *	0.169	0.152	0.162	0.132	0.090	0.009							
* Opistognathus whitehursti *	0.162	0.177	0.153	0.175	0.125	0.161	0.003						
* Opistognathus vicinus *	0.173	0.159	0.149	0.159	0.143	0.165	0.111	0.000					
* Opistognathus lonchurus *	0.151	0.170	0.176	0.170	0.142	0.173	0.139	0.165	n/a				
* Opistognathus aurifrons *	0.189	0.163	0.184	0.167	0.159	0.176	0.194	0.186	0.134	0.002			
(Caribbean Clade 2)													
* Opistognathus aurifrons *	0.181	0.171	0.186	0.178	0.160	0.180	0.184	0.193	0.128	0.032	0.006		
(Caribbean Clade 1)													
* Opistognathus aurifrons *	0.181	0.173	0.178	0.165	0.148	0.174	0.178	0.186	0.132	0.035	0.043	0.000	
(Fernando de Noronha)													
* Opistognathus aurifrons *	0.181	0.178	0.176	0.173	0.159	0.185	0.181	0.184	0.132	0.040	0.045	0.016	0.000
(Brazil mainland)													

## Key to Brazilian *Opistognathus*

**Table:** 

1	Anterior nostril with a simple cirrus on posterior margin; dorsal fin without a narrow dark margin; dorsal and anal fins with 0–1 anterior segmented rays unbranched distally	**2**
–	Anterior nostril a simple tube without a cirrus on posterior margin; dorsal fin with a narrow dark margin (blue in life); dorsal and anal fins with 6–10 anterior segmented rays unbranched distally	**5**
2	Adults with posterior end of maxilla rigid, not ending as thin, flexible lamina; dorsal-fin spines stiff, straight, the skin-covered tips usually pale and slightly swollen fleshy tabs; supramaxilla absent	**3**
–	Adults with posterior end of maxilla ending as thin, flexible lamina (slightly elongate in mature females and very elongate in males); dorsal-fin spines thin, flexible, usually curved distally, and tips without pale, slightly swollen tabs; supramaxilla present	**4**
3	Upper margin of subopercle a broad, fan-like flap; vomer with 1 tooth; premaxilla with two or more rows of teeth anteriorly; dorsal-fin segmented rays 15 or 16; caudal vertebrae 18	***O.thionyi* sp. n.**
–	Upper margin of subopercle not a broad, fan-like flap; vomer without teeth; premaxilla with one row of teeth anteriorly; dorsal-fin segmented rays 14; caudal vertebrae 17	***O.vicinus* sp. n.**
4	Dorsum with 5–6 dark blotches some extending on to base of dorsal fin; underside of upper jaw and adjacent membranes with two elongate dark stripes (males) or one smaller stripe (females); caudal fin without pale bands; caudal vertebrae 18	*** O. brasiliensis ***
–	Dorsum without dark blotches along base of dorsal fin; under side of upper jaw and adjacent membranes in adults with two dark blotches, the innermost one poorly developed (males) or dark blotches absent (females); caudal fin with two pale bands; caudal vertebrae 19	*** O. cuvierii ***
5	Dorsal- and anal-fin rays 12 or 13; dentary without large canines; caudal vertebrae 16	*** O. lonchurus ***
–	Dorsal- and anal-fin fin rays 14 or 15; dentary with large lateral canines; caudal vertebrae 17	*** O. aff. aurifrons ***

### Opistognathus thionyi sp. n.

Taxon classificationAnimaliaPerciformesOpistognathidae

http://zoobank.org/92465272-CF9F-48A3-B3C9-EEF22C594A26

**Figure 1.**
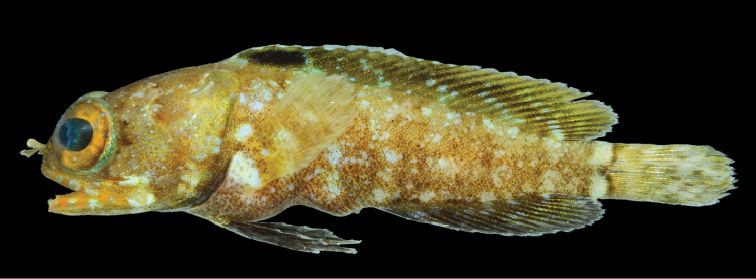
*Opistognathusthionyi*, CIUFES 2054, 27.5 mm SL, Dogaressa Seamount, Vitória-Trindade Chain. Photograph by Raphael M. Macieira.

**Figure 2.**
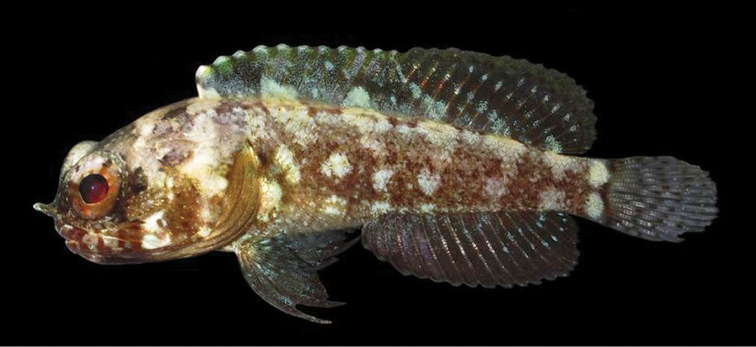
*Opistognathusthionyi*, NPM 5029, 38.4 mm SL, Enseada dos Portugueses, Trindade Island, Brazil. Photograph by Thiony Simon.

**Figure 3.**
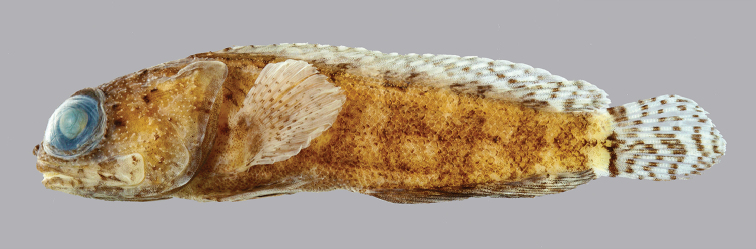
*Opistognathusthionyi*, CIUFES 2347, preserved holotype, 45.4 mm SL, male, Trindade Island, Brazil. Photograph by Zackary S. Randall.

**Figure 4.**
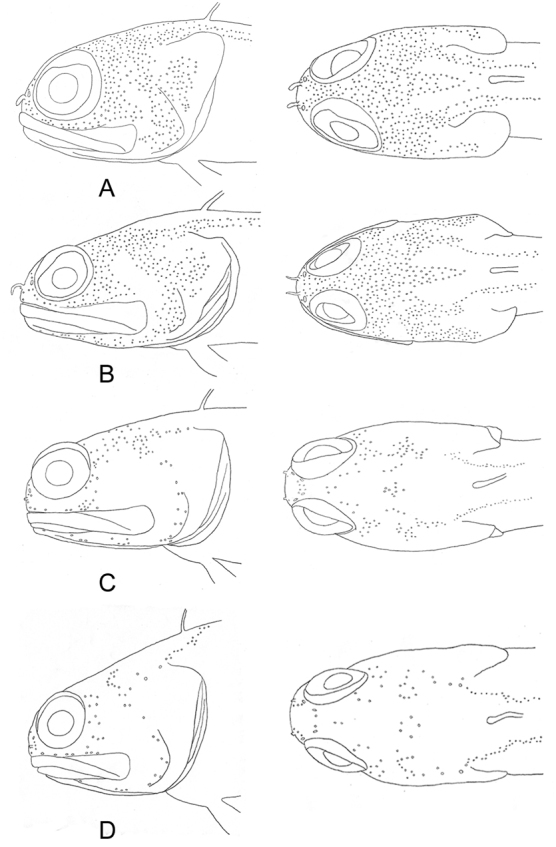
Cephalic sensory pores in selected species of *Opistognathus*. **A***O.thionyi*, 45.4 mm SL, holotype **B***O.vicinus*, NPM 5030, 47.4 mm SL, Brazil **C***O.lonchurus*, CIUFES 1426, 75.3 mm SL, Brazil **D**O.aff.aurifrons, CIUFES 1450, 57.0 mm SL, Brazil.

**Figure 5.**
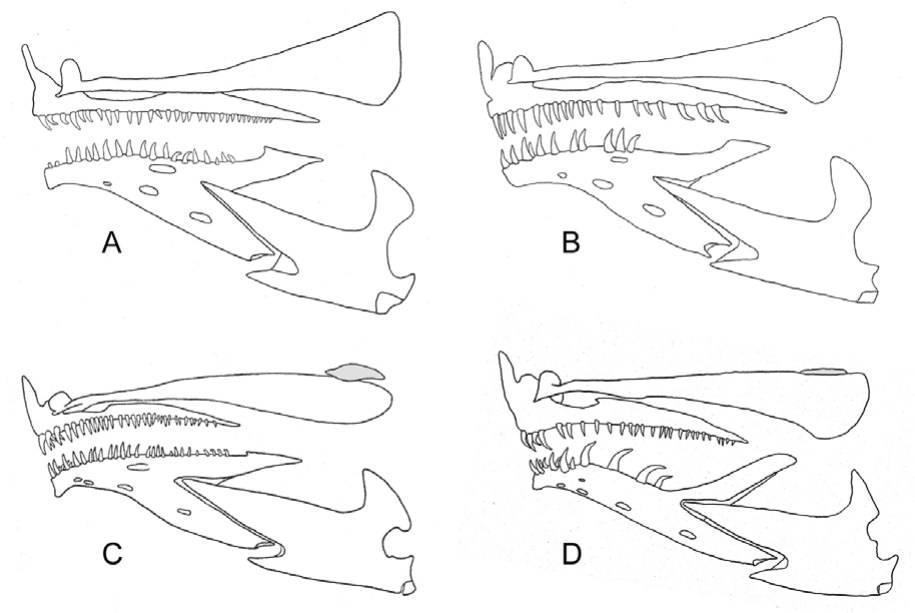
Jaws and dentition (supramaxilla shaded) in selected species of *Opistognathus*. **A***O.thionyi*, CIUFES 2393, 53.5 mm SL, Fernando de Noronha Archipelago, Brazil **B***O.vicinus*, NPM 5030, 47.4 mm SL, Brazil **C***O.lonchurus*, ANSP 126637, 75.0 mm SL, Gulf of Mexico **D**O.aff.aurifrons, ANSP 188905, 63.8 mm SL, Brazil.

Figures 1 , 2 , 3 , 4A , 5A , 7 ; Tables 1 , 2 , 3

Opistognathus sp.: Simon et al. 2013a: 2116, 2120 (undescribed species; listed and photograph); Pinheiro et al. 2015: 15, color fig. S. 38 (Dogaressa Seamount); Pinheiro et al. 2018: 10, Southwestern Atlantic (SWA) Endemic reef fishes – Annotated Checklist: 31.

#### Holotype.

CIUFES 2347, 45.4 mm SL, male, sandy rubble bottom at Praia do Lixo, Trindade Island, Brazil, 20°31'30"S, 29°19'20"W, 20 m, 20 February 2012, Thiony Simon and L.B.C. Xavier.

#### Paratypes.

(12 specimens 23.4–53.5 mm SL) all from Brazilian Province: MNRJ 51283 (1, 39.9*) and ZUEC 16914 (1, 44.9*), sandy rubble bottom at Praia do Lixo, Trindade Island, Brazil, 20°31'30"S, 29°19'20"W, 15 m, 18 February 2012, T. Simon and E.F. Mazzei; CIUFES 2344 (4, 23.4-32.7 & 32.5 C&S) and UF 239658 (1, 32.9), sandy rubble bottom at Praia do Lixo, Trindade Island, Brazil, 20°19'30"S, 29°19'20"W, 21 m, 21 February 2012, T. Simon and L.B.C. Xavier; AMNH 267140 (1, 32.5*), sandy rubble bottom at Praia do Lixo, Trindade Island, Brazil, 20°30'S, 29°20'W, 20 m, 20 February 2012, T. Simon and L.B.C. Xavier; CIUFES 2393 (1, 53.5), male, sandy rubble bottom at Portinho, Fernando de Noronha Archipelago, Brazil, 03°50'S, 32°24'W, 15 m, 11 July 2012, R.M. Macieira and H.T. Pinheiro; NPM 5029 (1, 38.4*), male, sandy rubble bottom at Enseada dos Portugueses, Trindade Island, Brazil, 20°31'30"S, 29°19'20"W, 25m, 8 August 2012, T. Simon and E.F. Mazzei; MZUSP 123868 (1, 30.9) and USNM 440401 (1, 35.3*), sandy rubble bottom at Praia do Lixo, Trindade Island, 20°31'30"S, 29°19'20"W, 17 m, 7 August 2012, T. Simon and L.B.C. Xavier.

#### Non-type material.

CIUFES 2054 (1, 27.5), sandy rubble bottom at Dogaressa seamount, Vitória-Trindade Chain, Brazil, 20°51'S, 33°40'W, 65 m, 12 April 2011, Expedição Cadeia Vitória-Trindade; CIUFES 2341-1 (1, 25.1), CIUFES 2341-2 (23.8) and CIUFES 2341-3 (1, 21.3), sandy rubble bottom at Praia do Lixo, Trindade Island, Brazil, 20°31'30"S, 29°19'20"W, 15 m, 18 February 2012, T. Simon and E.F. Mazzei; CIUFES 2346-3 (1, 29.2), sandy rubble bottom at Praia do Lixo, Trindade Island, Brazil, 20°31'30"S, 29°19'20"W, 20 m, 20 February 2012, T. Simon and L.B.C. Xavier.

#### Diagnosis.

A species of *Opistognathus* with the following combination of characters: anterior nostril a short tube with simple cirrus on posterior rim; maxilla rigid, not produced as a thin flexible lamina posteriorly; supramaxilla absent; subopercle with a broad, fan-like flap; vomer with 1 tooth; buccal area surrounding esophageal opening pale; body with 45–52 oblique body scale rows in longitudinal series; vertebrae 10+18; spinous dorsal fin with black blotch, when present, between spines 2–5. Body with five poorly defined irregular bands and sides sometimes with diagonal rows of pale spots smaller than eye diameter; when present, black blotch in spinous dorsal fin between spines 2–5; buccal area surrounding esophageal opening pale. This species is also easily distinguished from congeners by divergence in the mitochondrial gene COI, as specimens form a monophyletic group that differs from its closest relative (*O.maxillosus*) by an average of 9% (654 bp analyzed).

#### Description.

Morphometric data are given in Table 3 for the holotype and specimens indicated above by an asterisk; other comparative features are presented in Table 1. Where counts differ, those of the holotype are given first, followed in parentheses by those of the paratypes. Dorsal fin XI, 15 (15–16). Anal fin III, 15 (14–15, usually 15). Pectoral-fin rays 20 (19–20). Vertebrae: 10+18, last pleural rib on vertebra 10, epineurals 13–15. Supraneurals absent. Caudal fin: procurrent rays 5+5 (4-6+4-5); segmented rays 8+8, middle 12 branched, total elements 26 (24–26); hypural 5 absent. Gill rakers (number not increasing with increase in SL in adults) 9+19 (8–11+17–20=25–31).

**Table 3.** Morphometric data for holotype and six paratypes of *Opistognathusthionyi*.

Character	Holotype	Range	Mean	SD
Standard length (mm)	45.5	32.5–53.5	40.7	7.5
**Percentage of SL**				
Head length	38.6	34.8–37.6	36.5	0.99
Postorbital head length	22.6	19.6–22.0	21.0	0.91
Jaw length	22.3	21.2–22.1	21.7	0.41
Postorbital jaw length	6.2	3.9–7.7	5.7	1.40
Orbit diameter	13.8	11.8–14.2	12.9	0.80
Pelvic-fin length	23.7	24.2–25.3	24.7	0.50
Caudal-fin length	24.8	23.4–26.4	24.7	1.10
Body depth	20.1	19.0–21.8	19.9	1.13
Caudal peduncle depth	10.0	9.3–10.6	10.1	0.53
Predorsal length	36.0	32.2–35.4	33.9	1.24
Preanal length	56.9	58.1–61.2	59.7	1.13
Dorsal-fin length	62.6	62.6–67.4	64.6	1.79
Anal-fin length	31.9	29.3–34.9	32.8	2.16
**Percentage of HL**				
Postorbital head length	58.7	54.5–59.7	57.4	1.70
Jaw length	57.7	56.5–61.3	59.4	2.10
Postorbital jaw length	16.1	10.9–21.3	15.6	4.00
Orbit diameter	35.9	32.1–37.9	35.5	2.20
**Ratio**				
POJaw length/orbit diameter	0.45	0.30–0.61	0.44	0.13

Scales absent from head, nape, pectoral-fin base and breast; belly completely scaled, and sides fully scaled except for area above lateral line anteriorly. Body with 48 (45–52) oblique scale rows in longitudinal series. Lateral-line terminus below verticals between segmented dorsal-fin ray 3 (3–5). Anterior lateral-line pores relatively numerous and arranged in branched series along lateral-line tubes, all of which are embedded in skin. Mandibulo-preopercular pore positions all consisting of multiple pore series, except first two mandibular pore positions occupied by simple pores. Infraorbital pore positions consisting of multiple series that extend onto cheeks. Nape nearly to completely covered by sensory pores except for V-shaped naked area immediately in front of dorsal-fin origin (Figure 4A).

Anterior nostril positioned closer to posterior nostril than to dorsal margin of upper lip, and adults with a rounded cirrus that usually reaches anterior margin of orbit when depressed; height of cirrus 2.0–3.0 times maximum diameter of posterior nostril. Dorsal fin moderately low anteriorly, with posterior rays slightly longer; profile relatively uniform without noticeable change in fin height at junction of spinous and segmented rays. Dorsal-fin spines stiff and straight with pungent tips and in larger specimens the skin covered tips usually with pale, slightly swollen fleshy tabs. Segmented dorsal- and anal-fin rays all typically branched distally. Outermost segmented pelvic-fin ray not tightly bound to adjacent ray and interradial membrane strongly incised distally; tip of depressed pelvic fin in front of anal-fin origin. Upper margin of subopercle consisting of a broad, truncated flap (Figure 4A) and dorsalmost spine of opercle not noticeably elongate; posterior margin of preopercle distinct, with a well-developed groove dorsally. No papillae on inner surface of lips. Fifth cranial nerve passes over A1β section of adductor mandibulae muscle.

Upper jaw not sexually dimorphic, extending 0.45 (0.3–0.6) eye diameters behind orbit in specimens 32.5–53.5 mm SL; posterior end of maxilla rigid and truncate, without a thin flexible lamina; supramaxilla absent. Coronoid (ascending) process of articular slightly tilted backward and somewhat club-shaped with anterodorsal end bluntly pointed and posteroventral end bluntly rounded (Figure 5A). Premaxilla anteriorly with an outer row of stout teeth and an inner row of smaller, backward slanting teeth, some nearly horizontal; laterally teeth uniserial and becoming progressively smaller and more closely spaced. Dentary anteriorly with an outer row of stout teeth and an inner row of smaller, backward slanting teeth; laterally teeth uniserial and smaller but not progressively so. Vomer with 1 large tooth. Infraorbital bones tubular, with numerous openings for sensory canals; third infraorbital with a wide suborbital shelf. Postcleithra consisting of two well separated bones; dorsal postcleithrum an irregular elongate oval, narrowest ventrally, ventral postcleithrum rod-shaped with pointed ends.

**Color in life** (Figures 1, 2). Body coloration chestnut brown to dark brown with five very irregular and poor defined dark bands that extend onto base of dorsal fin; head sometimes with pale speckles on posterior half; upper jaw with a wide white band near posterior end; eyes reddish brown sometimes with narrow pale radiating bands; lips with alternating dark and pale bands; branchiostegal membranes dark, especially in mature males; dorsal fin light yellow sometimes with diagonal rows of pale spots and a black blotch, when present, between spines 2–5; anal fin with small pale spots; pelvic fins dark; pectoral fins speckled and a large white spot on pectoral-fin base; caudal fin with pair of pale basicaudal spots and fin rays with prominent black speckles or narrow bands.

**Preserved color** (Figure 3). As above except with white, brown, and black markings. Inner margin of maxilla posteriorly and adjacent membranes with dusky blotch. Buccal area surrounding esophageal opening pale.

#### Comparisons.

The Caribbean allopatric *Opistognathusmaxillosus* Poey, 1860 shares with *O.thionyi* the same subopercle shape but in addition to having more longitudinal body scale rows (69–85 vs. 45–52), the dark spot in the dorsal fin is always between spines 6–9 (vs. when present between spines 2–5), and the buccal area immediately surrounding the esophageal opening very dark (vs. pale). An updated description of *Opistognathusmaxillosus* is given in Smith-Vaniz (1997). The only other species of *Opistognathus* with a broad, fan-like subopercle are the eastern Pacific *O.galapagensis* Allen & Robertson, 1991 and *O.fossoris* Bussing & Lavenberg, 2003. In addition to other characters discussed by Bussing and Lavenberg (2003), both species differ from *O.thionyi* most notably in having the posterior end of maxilla ending as thin, flexible lamina (vs. end of maxilla rigid), more body longitudinal scale rows, 83–105 (vs. 45–52), most of nape immediately in front of dorsal-fin origin without cephalic pores (vs. almost completely covered with pores), and in having very different color patterns. Comparison of the six species of *Opistognathus* known from the Brazilian Province is given in Table 1.

#### Etymology.

The specific name honors our colleague and dear friend Thiony Simon (1985–2016), who passed away during preparation of this article. He collected most of the type material of the new species and dedicated his life to study and conservation of Brazilian reef ecosystems.

#### Distribution, habitat, and natural history.

**Figure 6.**
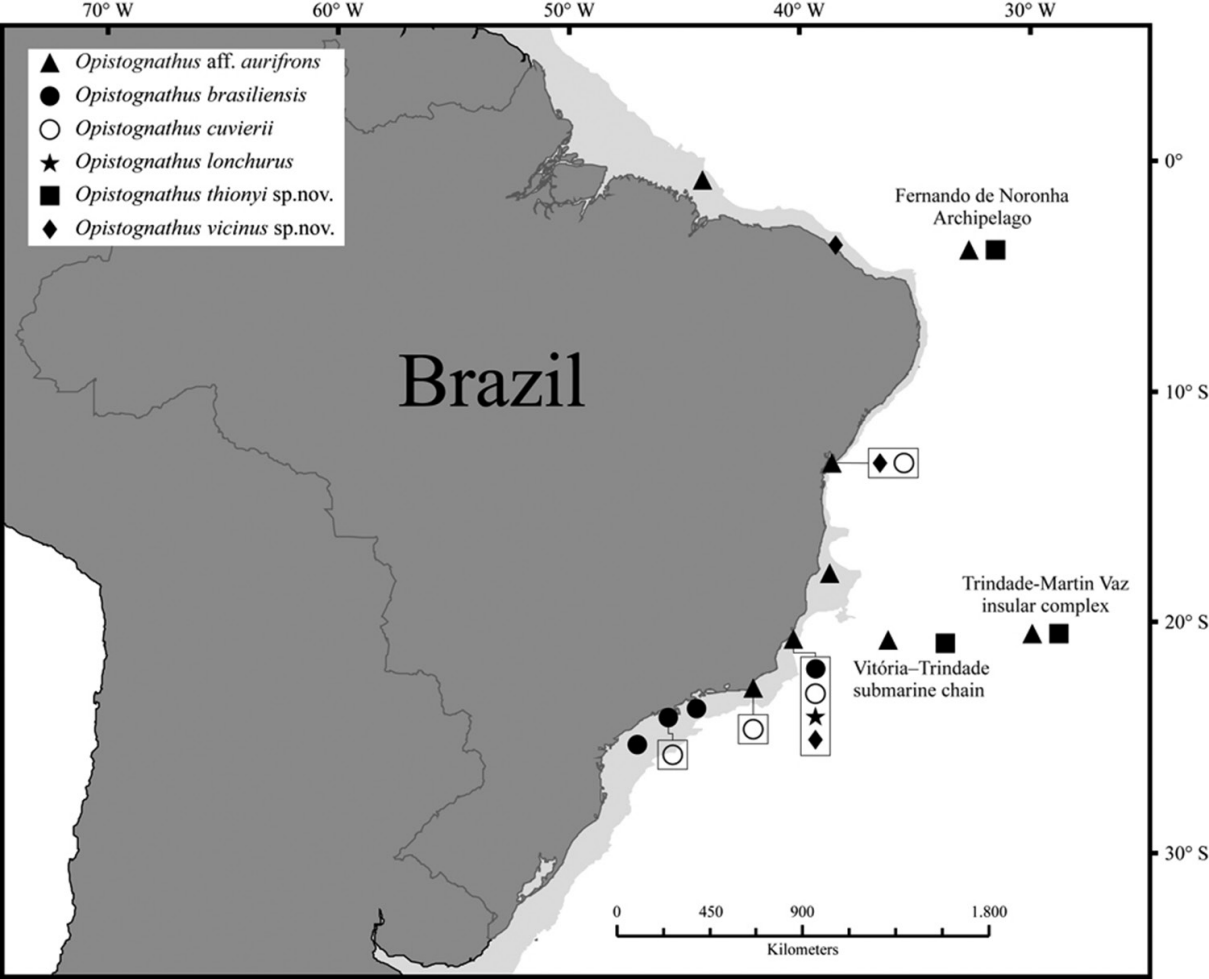
Distributions of *Opistognathus* species in Brazilian Province; light shaded areas indicate continental shelf.

**Figure 7.**
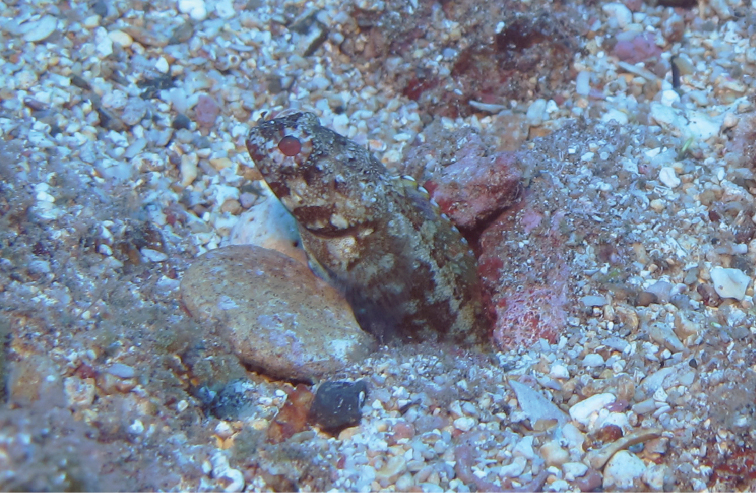
*Opistognathusthionyi*, in typical habitat, sandy rubble bottom in 13 m at Enseada dos Portugueses, Trindade Island, Brazil. Photograph by Thiony Simon.

*Opistognathusthionyi* is known only from three oceanic sites, Trindade Island, Dogaressa Seamount, and Fernando de Noronha Archipelago (Figure 6), and is an endemic species of the Brazilian Province (sensu Briggs and Bowen 2012 and Pinheiro et al. 2018). This species is possibly broadly distributed along the coast on the outer shelf, an area that is virtually unsampled. It has been recorded from 10–65 m, and found solitarily, always in small constructed burrows on sandy rubble bottoms (Figure 7). It feeds on small benthic organisms that live near the bottom (*e.g.*, small shrimps).

#### Conservation.

The conservation status of *Opistognathusthionyi* (cited as *Opistognathusmaxillosus* Poey, 1860 – unpublished data) has been assessed by the Ministério do Meio Ambiente/Instituto Chico Mendes de Conservação da Biodiversidade (MMA/ICMBio - Brazil) and listed as Least Concern. However, anthropogenic activities on oceanic marine ecosystems (i.e., seamount mining, fisheries, marine traffic, tourism, and human occupation of the islands), and the inadequate protection from these impacts currently provided by new Brazilian marine protected areas in the Vitória-Trindade Seamounts Chain (see Brasil 2018; Giglio et al. 2018; Magris and Pressey 2018), could threat the existence of Thiony’s jawfish in that part of its range in the near future.

### Opistognathus vicinus sp. n.

Taxon classificationAnimaliaPerciformesOpistognathidae

http://zoobank.org/42069A91-5C47-4651-B750-0689B05584E2

**Figure 8.**
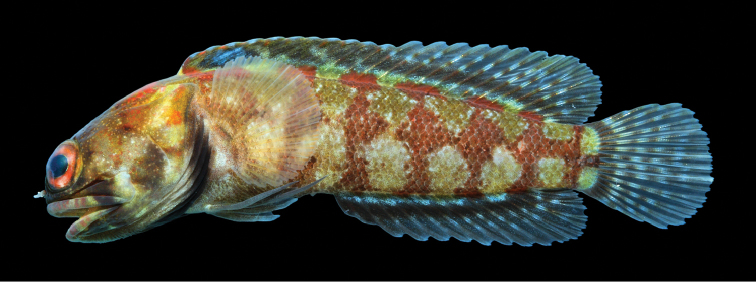
*Opistognathusvicinus*, CIUFES 0796, holotype, 43.0 mm SL, male, Ilha Rasa de Dentro, Guarapari, Espírito Santo, Brazil. Photograph by Raphael M. Macieira.

**Figure 9.**
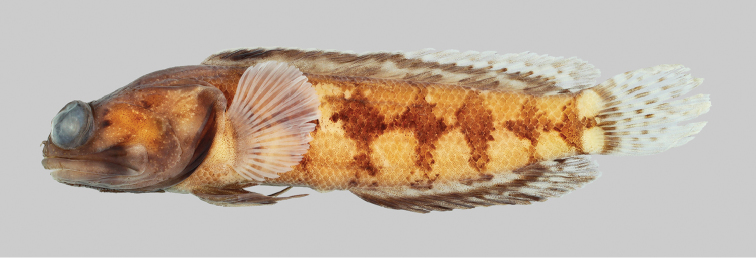
*Opistognathusvicinus*, NPM 5030, preserved paratype, 47.4 mm SL, Ilha Rasa, Guarapari, Espírito Santo, Brazil. Photograph by Zackary S. Randall.

Figures 4B , 5B , 8 , 9 ; Tables 1 , 2 , 4

Opistognathus whitehursti (Longley, 1927): Simon et al. 2013b (listed).

#### Holotype.

CIUFES 0796, 43.0 mm SL, male, Ilha Rasa de Dentro, Guarapari, Espírito Santo, 20°40'S, 40°21'W, 15m, 11 March 2008, R. M. Macieira and T. Simon.

#### Paratypes.

(12 specimens 17.0–47.4 mm SL) all from Brazil Province: UF 239659 (2, 27.9–31.0), taken with the holotype; CIUFES 0868 (1, 38.3*), Ilha Rasa de Dentro, Guarapari, Espírito Santo, 20°40'S, 40°21'W, 10 m, 29 January 2008, V.C. Brilhante; USNM 440402 (1, 38.6*), Ilha das Garças, Vila Velha, Espírito Santo, 20°36'S, 40°22'W, 30 March 2000, J.L. Gasparini; MZUSP 123869 (1, 38.9), MNRJ 51284 (1, 17.0), ZUEC 16915 (1, 42.5*) and AMNH 267141 (2, 21.7–38.9*), Ilha Escalvada, Guarapari, Espírito Santo, 20°42'S, 40°24'W, 21 m, 23 February 2010, R.M. Macieira, T. Simon and C.R. Pimentel; NPM 5030 (1, 47.4*), Ilha Rasa de Dentro, Guarapari, Espírito Santo, 20°40'S, 40°21'W, 31 October 2005, R.M. Macieira and J.-C. Joyeux; CIUFES 0467 (1, 26.2), Ilhas Rasas, Guarapari, Espírito Santo, 20°40'S, 40°21'W, 17 m, 5 December 2005, R.M. Macieira and J.-C. Joyeux; CIUFES 131797 (1, 36.0), Ilhas Rasas, Guarapari, Espírito Santo, 20°40'S, 40°21'W, 14 August 1989, J.L. Gasparini.

#### Diagnosis.

A species of *Opistognathus* with the following combination of characters: anterior nostril a short tube with simple cirrus on posterior rim; maxilla rigid, not produced as a thin flexible lamina posteriorly; supramaxilla absent; subopercle without a broad, fan-like flap; vomer without teeth; body with 43–47 oblique body scale rows in longitudinal series; vertebrae 10+17; sides with two rows of pale spots, each approximately diameter of eye. Body with six vertically irregular, evenly spaced bands, widest on mid-side, and two rows of six pale spots, each spot approximately diameter of eye; buccal area surrounding esophageal opening pale. This species is also easily distinguished from congeners by divergence in the mitochondrial gene COI, as specimens form a monophyletic group that differs from its closest relative (*O.whitehursti*) by an average of 11% (654 bp analyzed).

#### Description.

Morphometric data are given in Table 4 for the holotype and specimens indicated above by an asterisk; other comparative features are presented in Table 1. Where counts differ, those of the holotype are given first, followed in parentheses by those of the paratypes. Dorsal fin XI, 14. Anal fin II, 13 (II or III [anterior spine minute if III], 12–13, usually II, 13). Pectoral-fin rays 18 (17–18). Vertebrae: 10+17, last pleural rib on vertebra 10, epineurals 12–14. Supraneurals absent. Caudal fin: procurrent rays 4+4 (5–4+3–4); segmented rays 8+8, middle 12 branched, total elements 24 (23–25); hypural 5 absent. Gill rakers (number not increasing with increase in SL in adults) 7+16 (7–8+15–17=23–25).

**Table 4.** Morphometric data for holotype and six paratypes of *Opistognathusvicinus*.

Character	Holotype	Range	Mean	SD
Standard length (mm)	43.0	36.0–47.4	40.3	4.06
**Percentage of SL**				
Head length	37.1	34.1–37.5	35.7	1.11
Postorbital head length	24.0	21.2–24.2	22.8	1.23
Jaw length	20.5	17.2–21.2	19.9	1.57
Postorbital jaw length	7.8	6.3–8.9	7.7	0.88
Orbit diameter	10.2	10.1–11.3	10.9	0.57
Pelvic-fin length	23.4	23.1–25.6	24.6	1.04
Caudal-fin length	25.1	23.8–28.7	26.4	1.94
Body depth	22.5	19.5–22.2	20.9	0.94
Caudal peduncle depth	12.1	11.1–12.8	11.6	0.64
Predorsal length	34.8	31.3–36.6	34.3	1.80
Preanal length	53.8	55.6–58.8	57.4	1.16
Dorsal-fin length	71.0	60.9–68.1	64.5	2.35
Anal-fin length	36.4	31.1–36.5	34.5	1.94
**Percentage of HL**				
Postorbital head length	64.9	59.6–67.5	64.0	3.27
Jaw length	55.3	48.6–59.9	55.9	5.08
Postorbital jaw length	21.0	16.8–26.0	21.6	3.08
Orbit diameter	27.6	27.0–32.0	21.6	3.08
**Ratio**				
POJaw length/orbit diameter	0.76	0.62–0.88	0.71	0.09

Scales absent from head, nape, pectoral-fin base and breast; belly completely scaled, and sides fully scaled except for area above lateral line anteriorly. Body with 46 (43–47) oblique scale rows in longitudinal series. Lateral-line terminus below verticals between segmented dorsal-fin ray 1 (2–3). Anterior lateral-line pores relatively numerous and arranged in branched series along lateral-line tubes, all of which are embedded in skin. Mandibulo-preopercular pore positions all consisting of multiple pore series, except first two mandibular pore positions occupied by simple pores. Infraorbital pore positions consisting of multiple series that extend onto cheeks. Nape nearly to completely covered by sensory pores except for V-shaped naked area immediately in front of dorsal-fin origin (Figure 4B).

Anterior nostril positioned closer to posterior nostril than to dorsal margin of upper lip, and adults with a slender cirrus that reaches anterior margin of orbit when depressed; height of cirrus 2.0 times maximum diameter of posterior nostril. Dorsal fin moderately low anteriorly, with posterior rays slightly longer; profile relatively uniform without noticeable change in fin height at junction of spinous and segmented rays. Dorsal-fin spines stiff and straight and in larger specimens the skin covered tips usually with pale, slightly swollen fleshy tabs. Segmented dorsal- and anal-fin rays all typically branched distally. Outermost segmented pelvic-fin ray not tightly bound to adjacent ray and interradial membrane strongly incised distally; tip of depressed pelvic fin in front of anal-fin origin. Upper margin of subopercle oval-shaped without a broad, truncated flap (Figure 4B) and dorsalmost spine of opercle moderately elongate; posterior margin of preopercle distinct, with a well-developed groove dorsally. No papillae on inner surface of lips. Fifth cranial nerve passes over A1β section of adductor mandibulae muscle.

Upper jaw not sexually dimorphic, extending 0.76 (0.62–0.88) eye diameters behind orbit in specimens 36.0–47.4 mm SL; posterior end of maxilla rigid and truncate, without a thin flexible lamina; supramaxilla absent. Premaxilla with a single row of teeth, largest anteriorly becoming smaller and more closely spaced posteriorly, except in mature males posteriormost three or four teeth stouter and more strongly hooked than adjacent teeth. Dentary anteriorly with two rows of teeth, innermost smaller and slanted backwards; laterally teeth uniserial and larger than anterior teeth, posterior teeth of males larger and more strongly hooked than others. Vomer without teeth. Infraorbital bones tubular, with numerous openings for sensory canals; third infraorbital with a wide suborbital shelf. Postcleithra closely attached; dorsal postcleithrum an irregular elongate oval, narrowest ventrally where it overlaps head of ventral postcleithrum; ventral postcleithrum club-shaped, broadest dorsally and with a pointed ventral end.

**Color in life** (Figure 8). Background color of head and body brownish to reddish brown. Body with six vertically irregular and evenly spaced dark bands, widest on mid-side, that extend onto base of dorsal fin; two rows of six pale spots on sides, one along dorsal-fin base and the other along anal-fin base, each spot approximately diameter of eye; upper jaw with a wide dark band behind which is a white band at posterior end; the eyes are red; the lips with dark and pale bands; branchiostegal membranes dark; dorsal fin with dark stripe, widest anteriorly, along middle of fin, and a dark blue blotch between the second and fourth spine; pectoral fins are translucent; pelvic fins pale blue to dark or entirely pale; caudal fin with pair basicaudal spots bordered posteriorly by dark continuous band and remainder of fin vertical rows of dark spots or narrow bands.

**Preserved color** (Figure 9). Body with dark bands and large pale spots as above; other makings various shades of brown. Inner margin of maxilla posteriorly and adjacent membranes with brownish blotch, best developed in males. Buccal area surrounding esophageal opening pale.

**Figure 10.**
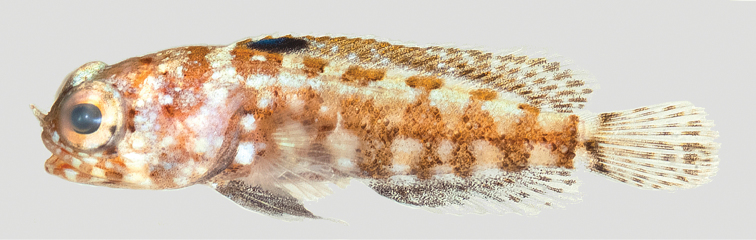
*Opistognathuswhitehursti*, USNM 396062, 22.0 mm SL, Saba Bank. Photograph by Jeffrey T. Williams.

**Figure 11.**
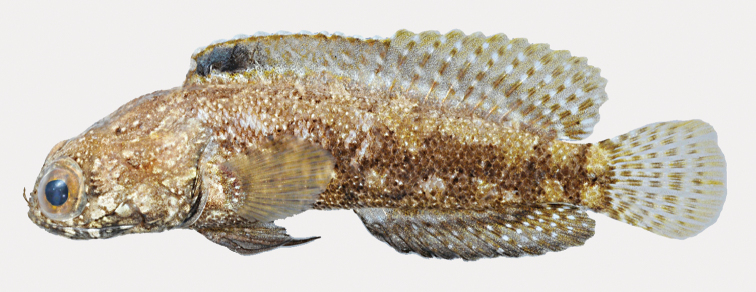
*Opistognathuswhitehursti*, UF 183105, 43.0 mm SL, St. Croix. Photograph by Howard L. Jelks.

#### Comparisons.

Genetic differences (see discussion in “Phylogenetic relationships of western Atlantic *Opistognathus*”), suggested that *Opistognathusvicinus* and the Caribbean *O.whitehursti* could be separate species despite their very similar appearance, including meristic values and sexually dimorphic premaxillary teeth (see Smith-Vaniz 1997: fig. 33). Initially we considered both species to be only “genovariants” sensu Victor (2015). However, *O.vicinus* lacks the small supramaxilla (see Smith-Vaniz 1997: fig. 32a) present in *O.whitehursti* and vomer without teeth (typically one or two teeth present in *O.whitehursti*). The color pattern of juveniles of *O.whitehursti* (Figure 10) is virtually identical to those of adult Brazilian fish. Unlike *Opistognathusvicinus*, adults of *O.whitehursti* usually have more reduced body bands (Figure 11) and the spinous dorsal-fin spot is often absent. As noted by Böhlke and Chaplin (1968: 486) for Bahamas fish, “The spot on the spinous dorsal fin is … blue in color in young, frequently missing or poorly defined in adults.” Thus, the combined differences in COI, adult coloration, and the lack of a supramaxilla and vomerine teeth support the recognition of *O.vicinus* as a distinct species. Comparison of the six species of *Opistognathus* known from the Brazilian Province is given in Table 1.

#### Etymology.

From the Latin *vicinus* (near, neighboring), referring to the allopatric distribution and sister-species phylogenetic relationship of the new species and the Caribbean *Opistognathuswhitehursti*.

#### Distribution and Habitat.

A Brazilian endemic (Figure 6), known from Ceará to Espírito Santo State but absent from oceanic islands. Common in coastal regions, in depths of 10–25 meters, associated with gravel-sand bottoms, near coral reefs and rocky areas. Feeds on small benthic organisms near the bottom (*e.g.*, small shrimps, crabs, and isopods).

#### Conservation.

The conservation status of this species [cited as *Opistognathuswhitehursti* (Longley 1927) – unpublished data] has been assessed by the Ministério do Meio Ambiente/Instituto Chico Mendes de Conservação da Biodiversidade (MMA/ICMBio - Brazil), and it was listed as Least Concern.

### Opistognathus brasiliensis

Taxon classificationAnimaliaPerciformesOpistognathidae

Smith-Vaniz, 1997

**Figure 12.**
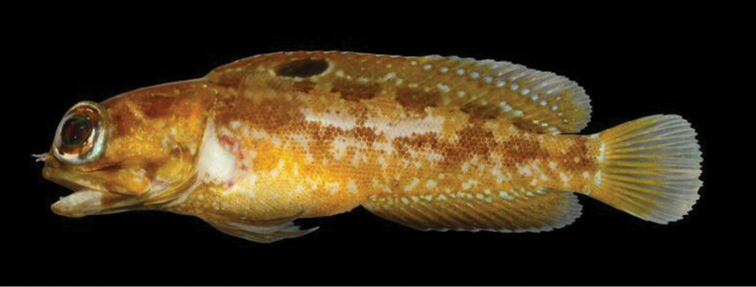
*Opistognathusbrasiliensis*, CIUFES 3361, 60.6 mm SL, female, Ilhas Escalvada, Guarapari, Espírito Santo, Brazil. Photograph by Raphael M. Macieira.

**Figure 13.**
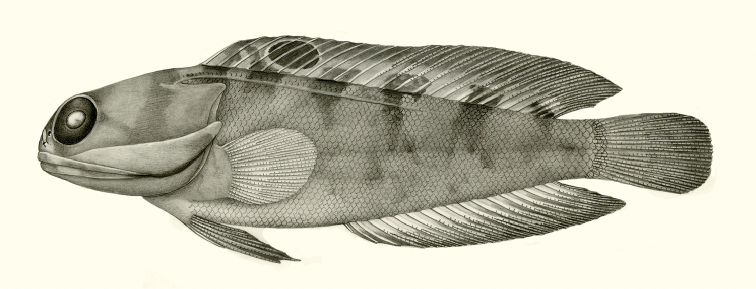
*Opistognathusbrasiliensis*, ANSP 141861, 122.5 mm SL, male, southeastern Brazil. Drawn by Jack R. Schroeder; after Smith-Vaniz (1997).

Figures 12 , 13 ; Tables 1 , 2

Opistognathus brasiliensis Smith-Vaniz, 1997: 1104, fig. 20 (original description; Alcatraces [misspelled Alcatrazes] Island: holotype MZUSP 13257); Carvalho-Filho 1999: 194 (abbreviated description); Menezes 2011: 42 (listed); Mincarone et al. 2017: 207 (listed); Pinheiro et al. 2018, Southwestern Atlantic (SWA) Endemic reef fishes – Annotated Checklist: 28.

#### Abbreviated description.

A species of *Opistognathus* with the following combination of characters: anterior nostril a short tube with simple cirrus on posterior rim; adults with posterior end of maxilla ending as thin, flexible lamina (slightly elongate in mature females and very elongate in males); supramaxilla present; subopercle without a broad, fan-like flap; most of nape without sensory pores; dorsal-fin spines thin, flexible, usually curved distally, and tips without pale, slightly swollen tabs; dorsal fin XI, 16 with all soft rays weakly branched distally; anal fin II, 15–16; body with 59–75 oblique scale rows in longitudinal series; vertebrae 10+18; supraneurals 2; gill rakers 9–11+23–24=33–36; spinous dorsal fin with black spot encircled by a very narrow white ring between spines 4–7 and dorsum with 5 or 6 dusky bands that extend onto base of dorsal fin; pelvic fins uniformly dark; underside of upper jaw and adjacent membranes in adults with two elongate dark stripes (males) or one smaller stripe (females) (Smith-Vaniz 1997, Figure 9c).

#### Material examined.

5 specimens (107.5–129 mm SL), including the holotype, cited in Smith-Vaniz (1997) plus the following additional material: CIUFES 1459 (1, 64.0), Ilhas das Garças, Vila Velha, Espírito Santo, 20°36'S, 40°22'W, 30 March 2000, J.L. Gasparini.

#### Distribution, habitat, and natural history.

A Brazilian endemic (Figure 6), known from Espírito Santo to São Paulo State, but absent from oceanic islands. A rare species only known from the type series collected in São Paulo’s coastal waters (trawled in 50–69 m) and two specimens from Guarapari Islands, Espírito Santo. Occurs in depths of 15–69 meters, associated with gravel and sand or silt and sand bottoms, near coral reefs and rocky areas.

#### Remarks.

In the diagnosis and description of *Opistognathusbrasiliensis*, Smith-Vaniz (1997) stated, in part, “buccal pigmentation consisting of a dark area widely surrounding esophageal opening …” versus area around esophageal opening pale in *O.cuvierii*. This reported distinction is no longer valid because in a recently examined female specimen of *Opistognathusbrasiliensis* (CIUFES 1459) the area around the esophageal opening is pale. Both species have black areas in front of each pharyngeal tooth patch that are separated by a pale median area.

#### Conservation.

The conservation status of this species has been assessed by the Ministério do Meio Ambiente/Instituto Chico Mendes de Conservação da Biodiversidade (MMA/ICMBio - Brazil), and it was listed as Data Deficient.

### Opistognathus cuvierii

Taxon classificationAnimaliaPerciformesOpistognathidae

Valenciennes, 1836

**Figure 14.**
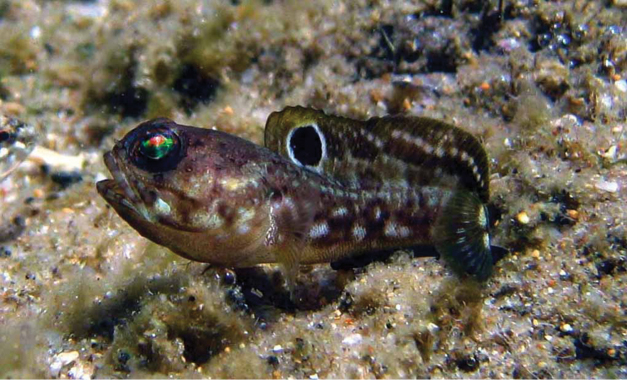
*Opistognathuscuvierii*, 80 mm TL estimated, Ilha dos Frades, Baía de Todos os Santos, Bahia, Brazil. Photograph by Cláudio L. S. Sampaio.

**Figure 15.**
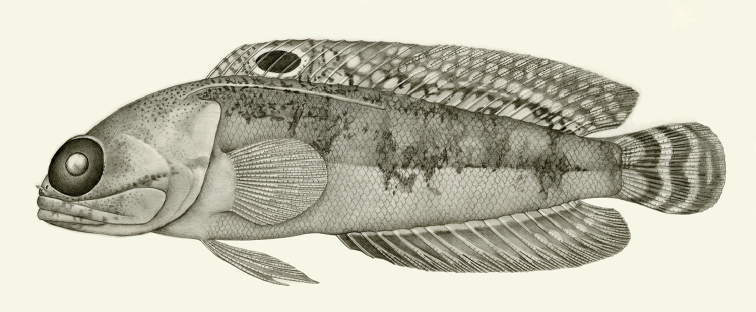
*Opistognathuscuvierii*, SU 52354, 102 mm SL, male, Bahia, Brazil. Drawn by Jack R. Schroeder; after Smith-Vaniz (1997).

Figures 14 , 15 ; Tables 1 , 2

Opisthognathus [sic] cuvierii Valenciennes in Cuvier and Valenciennes 1836: 504, color pl. 343 (original description; Bahia: holotype MNHN A. 2108); Roux 1964: 413, pl. 10 (listed; original illustration of holotype reproduced).
Opistognathus cuvierii : Menezes and Figueiredo 1985: 42, fig. 47 (description); Smith-Vaniz 1997: 1106, fig. 21 (description); Pinheiro et al. 2018, Southwestern Atlantic (SWA) Endemic reef fishes – Annotated Checklist: 28–29, color fig. 18.
Opistognathus cuvieri : Roux 1973: 151 (description); Carvalho-Filho 1999: 194 (abbreviated description and occurrence to São Paulo); Mincarone et al. 2017: 207 (listed).

#### Abbreviated description.

A species of *Opistognathus* with the following combination of characters: anterior nostril a short tube with simple cirrus on posterior rim; adults with posterior end of maxilla ending as thin, flexible lamina (slightly elongate in mature females and very elongate in males); supramaxilla present; subopercle without a broad, fan-like flap; most of nape without sensory pores; dorsal-fin spines thin, flexible, usually curved distally, and tips without pale, slightly swollen tabs; dorsal fin XI, 16, with all soft rays weakly branched distally; anal fin II, 16; body with 60–72 oblique scale rows in longitudinal series; vertebrae 10+19; supraneurals 1 or 2; gill rakers 9–11+20–23=30–35; spinous dorsal fin with an ocellus between spines 3–7, otherwise dorsal fin with rows of pale spots and dorsum without 5 or 6 dusky bands that extend onto base of dorsal fin; pelvic fins uniformly dark; caudal fin dark with two pale bands; underside of upper jaw and adjacent membranes in adults with two dark blotches, the innermost one poorly developed (males) (Smith-Vaniz 1997: fig. 9d), or these blotches absent (females); buccal pigmentation consisting of a dark blotch on either side of esophageal opening widely separated by pale median area that continues between upper pharyngeal tooth patches (Smith-Vaniz 1997: fig. 13c).

#### Material examined.

5 specimens (80.5–11.5 mm SL), including the holotype, cited in Smith-Vaniz (1997).

#### Distribution, habitat, and natural history.

A Brazilian endemic (Figure 6), known from Bahia to São Paulo but absent from oceanic islands. Distributed in coastal regions, in depths between 1–35 meters, associated with gravel and sand or silt and sand bottoms, near coral reefs and rocky areas. This species has been observed resting on the bottom (Figure 14) or in burrows (Pinto 1970).

#### Conservation.

The conservation status of this species has been assessed by the Ministério do Meio Ambiente/Instituto Chico Mendes de Conservação da Biodiversidade (MMA/ICMBio - Brazil) and it was listed as Least Concern.

### Opistognathus lonchurus

Taxon classificationAnimaliaPerciformesOpistognathidae

Jordan & Gilbert, 1882

**Figure 16.**
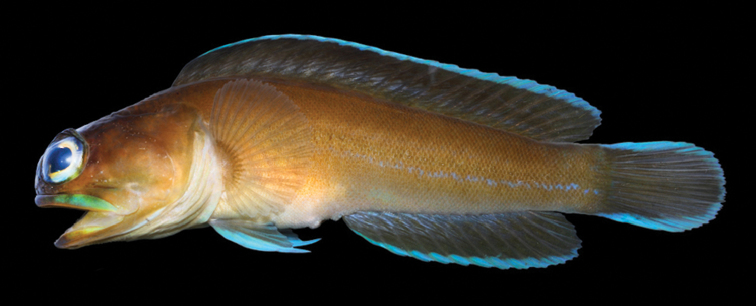
*Opistognathuslonchurus*, CIUFES 2361, 81.2 mm SL, female, Ilhas Escalvada, Guarapari, Espírito Santo, Brazil. Photograph by Raphael M. Macieira.

Figures 4C , 5C , 16 ; Tables 1 , 2

Opisthognathus [sic] lonchurus Jordan & Gilbert, 1882: 290 (original description; snapper banks off Pensacola, Florida: holotype USNM 30864); Böhlke and Thomas 1961: 514–515, Table 2.
Opistognathus lonchurus : Floeter and Gasparini 1999: 58 (Brazil occurrence); Carvalho-Filho 1999: 194 (abbreviated description).

#### Abbreviated description.

A species of *Opistognathus* with the following combination of characters: anterior nostril a short tube without a cirrus on posterior rim; posterior end of maxilla rigid, not produced as a thin flexible lamina; supramaxilla present; subopercle without a broad, fan-like flap; most of nape without sensory pores (Figure 4C); dorsal fin XI, 12–13, with spines thin and flexible, usually curved distally, tips without pale, slightly swollen tabs, and anterior 5–8 soft rays unbranched; dorsal fin sexually dimorphic, fin rays distinctly higher in large males; anal fin III, 12, with 7–10 anterior rays unbranched; outermost segmented pelvic-fin ray tightly bound to adjacent ray and interradial membrane not incised distally; body with 63–87 oblique scales in longitudinal series; vertebrae 10+16; supraneurals 1; gill rakers 13–17+22–28=35–45; dentary without large canines (Figure 5C). Head and body brown to greenish-tan; upper lip blue; body with two narrow blue stripes on side; distal margins of dorsal and anal fins with narrow blue stripe; outer margin of caudal fin and outer ray of pelvic fin blue.

#### Material examined.

3 specimens (75.3–81.2 mm SL) from Brazil and 46 specimens (27–122 mm SL) from the Caribbean. **Brazil**: CIUFES 1426 (1, 75.3), Ilhas Rasas, Guarapari, Espírito Santo, 20°40'S, 40°21'W, 26 February 2000, D.A. Jório and J.L. Gasparini; CIUFES 2361 (1, 81.2), gravid female, Ilhas Escalvada, Guarapari, Espírito Santo, 20°41'S, 40°24'W, 23 m, 30 February 2012, R.M. Macieira and J.-C. Joyeux; ZUEC 3160 (1), Mar do Bom Nome, Espírito Santo, 21°09'S, 40°30'W, 30 m, 8 December 1996, J.L. Gasparini. **Caribbean**, only abbreviated localities, standard lengths and depths given: ANSP 83680 (1, 88), Haiti; ANSP 126637 (1, 75 C&S), off Mobile, Alabama, 35 m; ANSP 134237 (1, 96), Puerto Rico, 49 m; ANSP 138138 (1, 86), Puerto Rico, 52 m; ANSP 138362 (1, 93), Puerto Rico, 52 m; ANSP 140955 (5, 77-97), Puerto Rico, 50 m; ANSP 142700 (1, 107), Puerto Rico; 14 m; ANSP 174157 (1, 83), Gulf of Mexico, 74 m; ANSP 177885 (2, 82-97), Gulf of Mexico, 63 m; ANSP 136545 (1, 92), off South Carolina, 60 m; CAS 1863 (1, 84), CAS 29250 (1, 84) and CAS 36683 (1, 95), Pensacola, Florida; FSBC 1706 (2, 85.5-94), 32°01'N, 79°24'W, R/V Silver Bay sta. 1788, 64–82 m; FMNH 79582 (1, 76), off Guyana, 49–55 m; FSBC 3314 (1, 107), off St. Petersburg, Florida, 47 m; FSBC 3324 (1, 93), Gulf of Mexico, 37 m; FSBC 12575 (1, 99), Florida Keys, 76–80 m; MCZ 52103 (2, 112-122), 55 m; Dry Tortugas, 55 m; SIO 70–186 (2, 77–97), Florida Keys, 40 m; SIO 70–186 (2, 76–96), Florida Keys, 40 m; SIO 70–224 (1, 47), Florida Keys, 38 m; UF 186218 (1, 80), Gulf of Mexico; UF 186226 (1, 89), Gulf of Mexico, 61 m; UF 186239 (1, 102), Gulf of Mexico; UF 218742 (1, 28), Florida, 38 m; UF 203991 (1, 90), off Guyana, 55–60 m; UF 219018 (1, 35), Florida, 40 m; UF 238298 (1, 196), Gulf of Mexico; USNM 31903 (1, 64), Pensacola, Florida; USNM 34976 (1, 69), holotype of *Gnathypopsmystacinus*), Pensacola, Florida; USNM 117035 (1, 27), Dry Tortugas; USNM 217802 (1, 96), off Colombia, 73 m; USNM 217083 (1, 106) off Colombia, 84 m; USNM 30712 (1, 104), Pensacola, Florida; USNM 358160 (1, 68), Gulf of Mexico, 62 m; USNM 358161 (1, 73), Gulf of Mexico, 67 m.

#### Distribution, habitat, and natural history.

**Figure 17.**
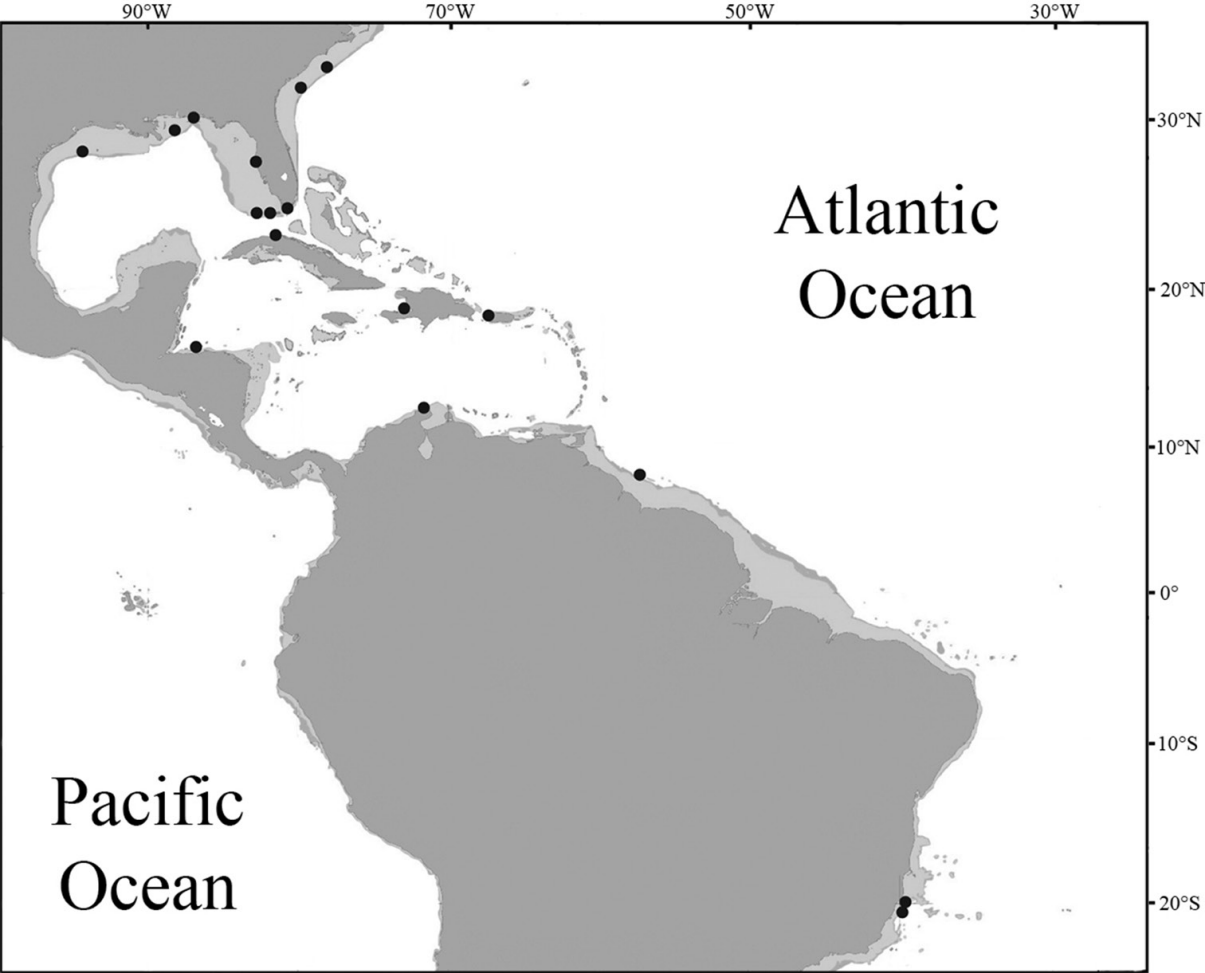
Distribution of *Opistognathuslonchurus*, including a few records from Robertson and Van Tassell (2015).

South Carolina, Gulf of Mexico, Greater Antilles and northern South America to Brazil (Figures 6, 17) in about 15–90 m. In the Brazilian Province known from the northern coast to Espírito Santo State but absent from oceanic islands. Occur in depths of 10–91 meters, in rubble-sand bottoms near coral reefs and rocky areas. It has been observed in burrows or resting on the bottom.

#### Conservation.

The conservation status of this species was assessed by the International Union for Conservation of Nature (IUCN) and listed as Lease Concern (Smith-Vaniz et al. 2015). The conservation status of this species was also assessed by the Ministério do Meio Ambiente/Instituto Chico Mendes de Conservação de Biodiversidade (MMA/ICMBio – Brazil) and listed as Least Concern.

### Opistognathus aff. aurifrons

Taxon classificationAnimaliaPerciformesOpistognathidae

Jordan & Thompson, 1905

**Figure 18.**
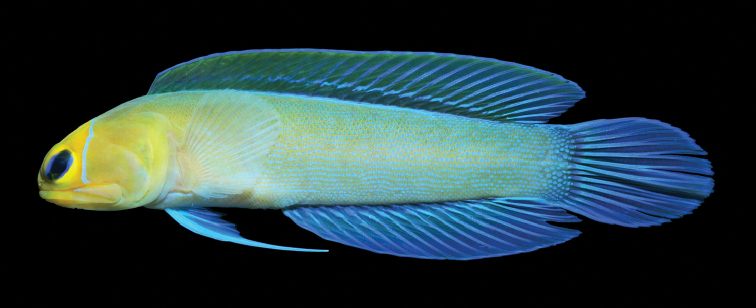
Opistognathusaff.aurifrons, CIUFES 0795-1, 61.8 mm SL, female, Ilhas Escalvada, Guarapari, Espírito Santo, Brazil. Photograph by Raphael M. Macieira.

**Figure 19.**
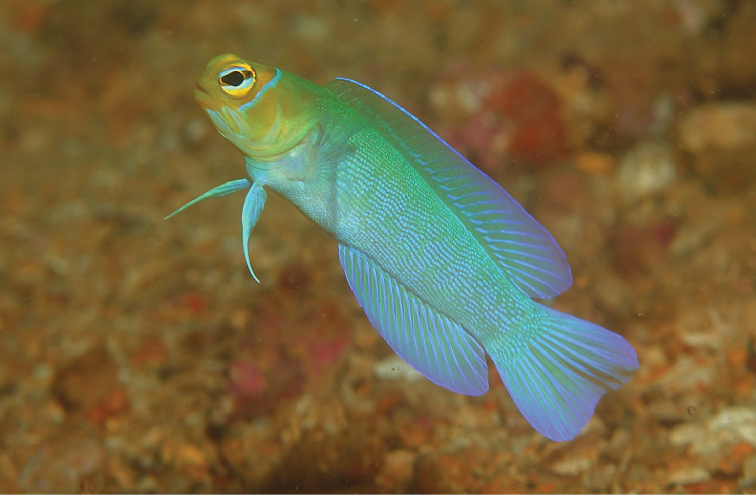
Opistognathusaff.aurifrons, adult, Bellucia shipwreck, Guarapari, Espírito Santo, Brazil. Photograph by Raphael M. Macieira.

**Figure 20.**
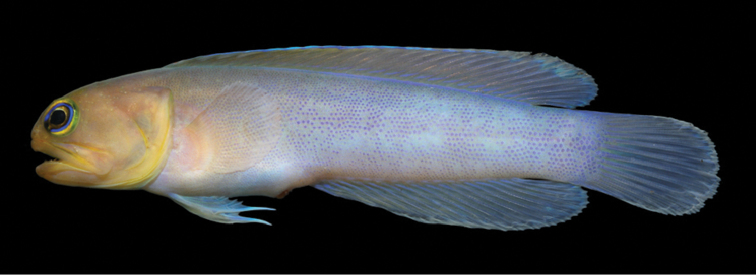
Opistognathusaff.aurifrons, CIUFES 2551, 69.2 mm SL, Fernando de Noronha Archipelago, Brazil. Photograph by Raphael M. Macieira.

**Figure 21.**
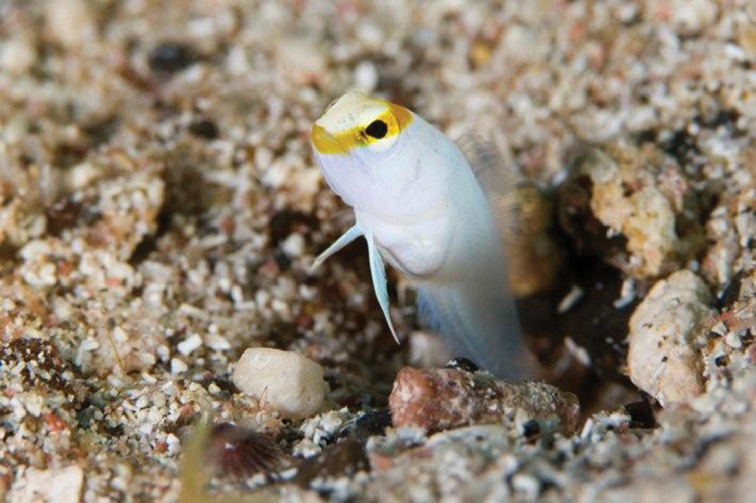
Opistognathusaff.aurifrons, juvenile, Fernando de Noronha Archipelago, Brazil. Photograph by João P. Krajewski.

Figures 4D , 5D , 18 , 19 , 20 , 21 ; Tables 1 , 2

Opistognathus aurifrons Jordan & Thompson, 1905: 252, fig. 4 (original description; Garden key, Dry Tortugas, Florida); Moura et al. 1999: 517 (Brazilian occurrence); Pereira-Filho et al. 2015: 69–70 (Listed, Fernando de Noronha Archipelago); Stocco and Joyeux 2015: 6 (ichthyoplankton, Trindade Island).
Opistognathus sp. Rocha and Rosa 2001: 990 (listed; Manuel Luiz Marine State Park, Brazil); Gasparini and Floeter 2001: 1644 (listed; Trindade Island); Rocha 2002: 477, unnumbered color fig. (Parcel Manuel Luiz, Brazil); Menezes et al., 2003: 78; Moura and Sazima 2000: 482 (listed); Sampaio and Nottingham 2008: 171 (abbreviated description).
Opistognathus sp. 1 Carvalho-Filho 1999: 193, color fig. 185; Pinheiro et al. 2018, Southwestern Atlantic (SWA) Endemic reef fishes – Annotated Checklist: 29–30, color fig. 19 (Fernando de Noronha Archipelago).
Opistognathus sp. 2 Smith-Vaniz 1997: 1096 (in identification key); Simon et al. 2013a: 2120 (listed); Pinheiro et al. 2018, Southwestern Atlantic (SWA) Endemic reef fishes – Annotated Checklist: 30–31, color fig. 20.
Opistognathus aff. aurifrons Jordan & Thompson: Feitoza et al. 2005: 732 (Brazilian Province in 35–54 m); Simon et al. 2013b: 63 (listed); Pinheiro et al. 2015: 15, color fig. S.37 (Trinidade Island and Dogaressa Seamount).

#### Abbreviated description (Brazilian specimens only).

A species of *Opistognathus* with the following combination of characters: anterior nostril a short tube without a cirrus on posterior rim; posterior end of maxilla rigid, not produced as a thin flexible lamina; supramaxilla present; dorsal-fin spines thin, flexible, usually curved distally, and tips without pale, slightly swollen tabs; subopercle without a broad, fan-like flap; most of nape without sensory pores (Figure 4D); dorsal fin XI, 14–15, with 6–11 anterior rays unbranched distally; anal fin III, 14–15, with 7–10 anterior rays unbranched; outermost segmented pelvic-fin ray tightly bound to adjacent ray and interradial membrane not incised distally; scales in longitudinal series 66–76; vertebrae 10+17; supraneurals absent; gill rakers 15–20+26–32=41–51; dentary with large lateral canines (Figure 5D). Life color of adults of the two different color morphs as in Figs 18–20 and discussed below in Remarks.

#### Distribution.

As provisionally recognized, this species is known only from the Brazilian Province including continental localities from the State of Maranhão (0°53'S, 44°17'W) south to Armação de Búzios (22°45'S, 41°59'W) in the State of Rio de Janeiro and oceanic sites of the Vitória-Trindade Seamounts Chain and Fernando de Noronha Archipelago (Figure 6). It is common in coastal regions, in depths of 10–30 meters, and in oceanic sites of 10–65 meters, associated with rubble and sand bottoms, near coral reefs and rocky areas. Its behavior and life history are similar of that of the Caribbean *O.aurifrons* (Colin 1971; Colin 1973). In this species the burrows are constructed on sandy or rubble bottoms, near reefs, using small stones, shell or coral fragments. They feed on zooplankton while hovering at a small distance over the substrate during quick incursions and generally retreat tail first into the burrow.

#### Material examined.

33 specimens (30.4–73.7 mm SL) all from Brazilian Province. **Mainland localities**: ANSP 188905 (2, 35.4–63.8 C&S), Naufrágio Bellucia (shipwreck), off Guarapari, Espírito Santo, 20°40'S, 40°21'W, 24.6 m, 29 August 2008, A. Carvalho-Filho, R.M. Macieira and C.R. Pimental; CAS 238006 (3, 53.2–57.5), Ilha Escalvada, Guarapari, Espírito Santo, 20°42'S, 40°24'W, 30 March 2012, L.A. Rocha; CAS 238007 (4, 58.8–70.3), Ilha Escalvada, Guarapari, Espírito Santo, 20°42'S, 40°24'W, 15 February 2012, L.A. Rocha; CIUFES 0795 (4, 24.0–66.9), Naufrágio Bellucia (shipwreck), off Guarapari, Espírito Santo, 20°40'S, 40°21'W, 27.0 m, 11 March 2008, J.-C. Joyeux, R.M. Macieira and V.C. Brilhante; CIUFES 1450 (2, 57.1–73.7 C&S), Ilhas Rasas, Guarapari, Espírito Santo, 20°40'S, 40°22'W, 15 m, 14 August 1999, J.L. Gasparini; MZUSP 44937 (1, 52.4), Ilhas Rasas, Guarapari, Espírito Santo, 20°40'S, 40°21'W, January 1992, J.L. Gasparini; MZUSP 46191 (1, 67.5), male, Cabo Frio, Rio de Janeiro, 22°53'S, 42°00'W, March 1991, A. Carvalho-Filho; MZUSP 46541 (1, 61.8), gravid female, Vitória, Espírito Santo, 20°19'S, 40°21'W, December 1990, A. Carvalho-Filho; MZUSP 52271 (2, 35.0–42.8), Ilha Sueste, Abrolhos Archipelago, 17°58'S, 38°41'W, 11 January 1997, I. Sazima, C. Sazima, J.L. Gasparini and R.L. Moura; MZUSP 52453 (1, 46.9) and UF 191039 (3, 30.4–51.6), Ilhas Rasas, Guarapari, Espírito Santo, 20°40'S, 40°22'W, 22 April 1992, D.A. Jório; ZUEC 3105 (1, 72.6), Ilhas Rasas, Guarapari, Espírito Santo, 20°40'S, 40°22'W, 18 m, 1 June 1996, D.A. Jório; ZUEC 2739 (1, 61.6), gravid female, Ilha Escalvada, Guarapari, Espírito Santo, 20°42'S, 40°24'W, 16 m, July 1995, D.A. Jório and J.L. Gasparini; UFPB 4047 (5, 52.4–67.5), Três Ilhas Archipelago, Guarapari, Espírito Santo, 20°36'S, 40°22'W, 1 December 1997, J.L. Gasparini. **Fernando de Noronha Archipelago**: CIUFES 2550 (1, 58.4) and CIUFES 2551 (1, 69.2), Cabeço Submarino, 03°52'S, 32°25'W, 19.6 m, 8 April 2013, R.M. Macieira and T. Simon.

#### Remarks.

**Figure 22.**
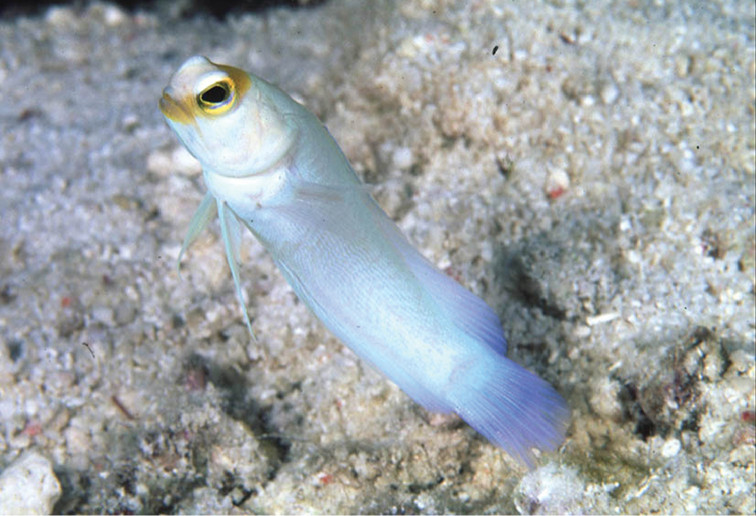
*Opistognathusaurifrons*, juvenile, Bonaire. Photograph by Scott Michael.

Brazilian specimens of Opistognathusaff.aurifrons (n=28) differ from Caribbean *O.aurifrons* (n=292) in consistently having 17 vs. 16 caudal vertebrae. Brazilian fish are represented by two allopatric and slightly different genetic populations (see discussion below in “Phylogenetic relationships of western Atlantic *Opistognathus*”). Mainland and Vitória-Trindade Seamounts Chain specimens have long pelvic fins that when depressed extend at least to the anal-fin origin (25.7–38.2% SL, mean 30.4%, in 22 specimens 30.4–74.8 mm SL) and in fresh adult specimens the top of the head is yellow, bordered posteriorly by a narrow blue band extending from slightly behind the eye to upper jaw and across the nape; remainder of the head and body greenish-yellow to bluish-yellow (Figures 18–19). Populations from the Fernando de Noronha Archipelago differ in having short pelvic fins that do not extend to the anal-fin origin (20.1–20.7% SL in 2 specimens 58.4–69.2 mm SL) and in fresh adults the head is pale tan-yellow, the body is pale grey-blue, and both are uniformly colored (Figure 20). To further complicate the situation, uncollected juveniles from Fernando de Noronha (Figure 21) and Bonaire (Figure 22) have identical life coloration consisting primarily of a white head crossed by diagonal brown-orange stripe about width of pupil extending from chin, through eye and across nape; body and fins pale grey. In preservation, adults from Tobago (USNM 317005) have the same head color pattern and long pelvic fins as those of the Brazilian mainland population but differ in having 16 caudal vertebrae. Unfortunately, life coloration was unrecorded and tissue samples were not obtained. A color photograph taken by Les Wilkes of a “Bluebar jawfish” from St. Vincent, Lesser Antilles (13°15'N, 61°12'W) also looks like mainland Brazilian adults. Overall, there are morphological characters that collectively differentiate the two Brazilian clades from the Caribbean haplotypes (*i.e*., vertebral counts) and from each other (i.e., pelvic fin length, but note the low sample size), but we know of no consistent phenotypic characters that differentiate the two Caribbean haplotypes from each other. The type locality for *O.aurifrons* is Dry Tortugas, Florida, making it likely that if the main groups here do indeed represent distinct species, the group containing specimens from Florida (green in Figure 24) represents the true *O.aurifrons* and the others new species. However, we refrain from making taxonomic changes pending a more thorough analysis comparing multiple genetic loci, and live coloration/morphology of vouchered specimens.

Depending on the locality, adults of Caribbean *Opistognathusaurifrons* may have relatively short or long pelvic fins, color patterns not found in Brazilian populations or that duplicate them.

#### Conservation.

The conservation status of Brazilian populations of this species has been assessed by the Ministério do Meio Ambiente/Instituto Chico Mendes de Conservação de Biodiversidade (MMA/ICMBio – Brazil) and listed as Least Concern.

## Phylogenetic relationships of western Atlantic *Opistognathus*

**Figure 23.**
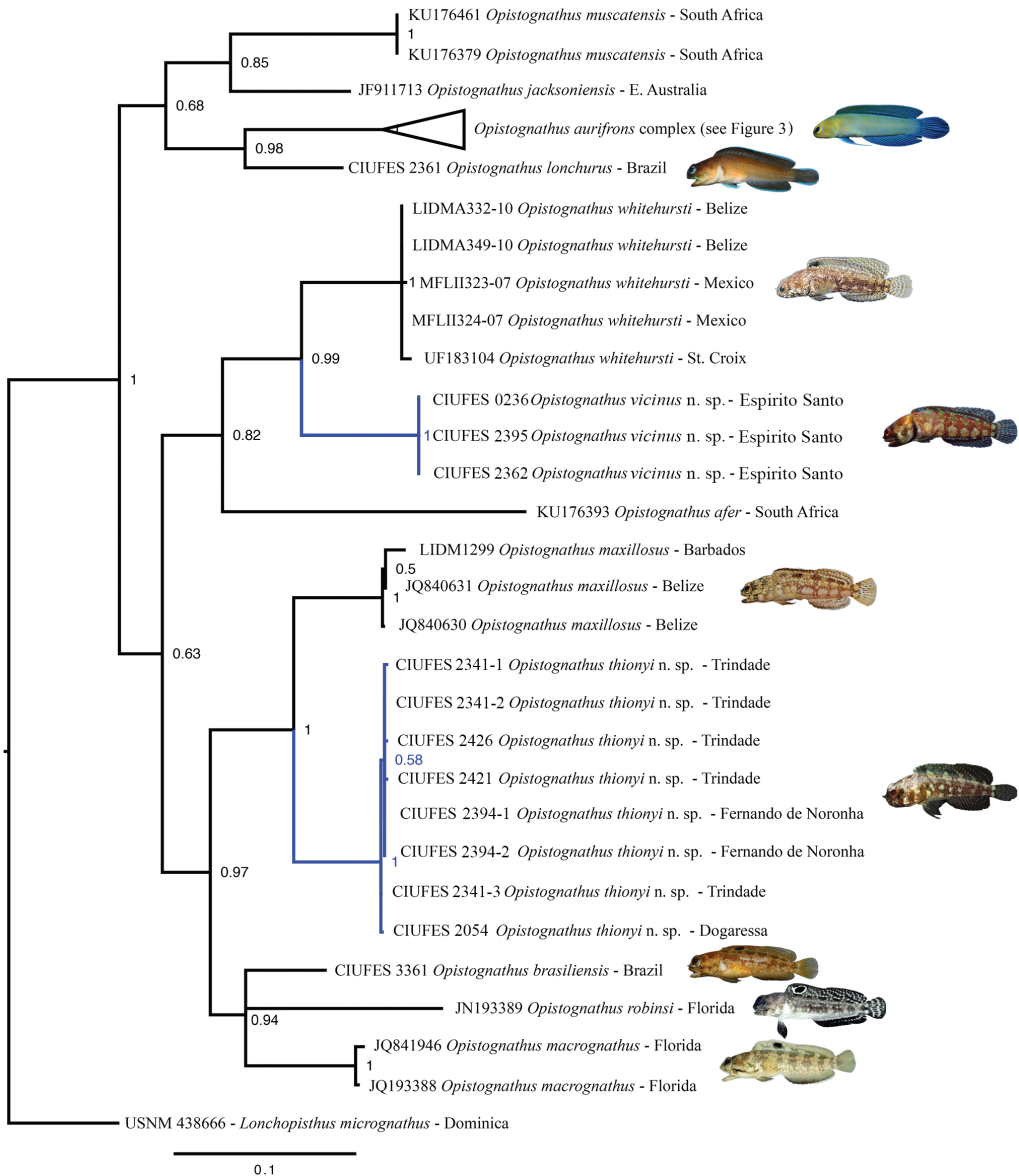
Bayesian inference phylogeny of western Atlantic *Opistognathus* based on COI data. Support values are Bayesian posterior probabilities. For clarity the clade containing *Opistognathusaurifrons* is collapsed (see Figure 24 for this clade expanded). Scale bar units are expected number of substitutions per site.

The molecular phylogenies inferred from the COI data using ML and Bayesian inference were very similar in topology (Figure 23); also see supporting information for ML trees. Most basal nodes were not well supported in either analysis, indicating that additional slower evolving genetic markers are needed to better resolve the phylogeny. Several clades containing western Atlantic *Opistognathus* were well supported, including a clade containing *O.whitehursti* and *O.vicinus*, another clade containing *O.lonchurus* and *O.aurifrons*, and a clade containing *O.maxillosus*, *O.thionyi*, *O.brasiliensis*, *O.robinsi*, and *O.macrognathus*. In the latter clade, the new species *O.thionyi* was resolved as a sister species to *O.maxillosus*, with that clade being sister to a clade containing *O.robinsi*, *O.brasiliensis*, and *O.macrognathus*.

**Figure 24.**
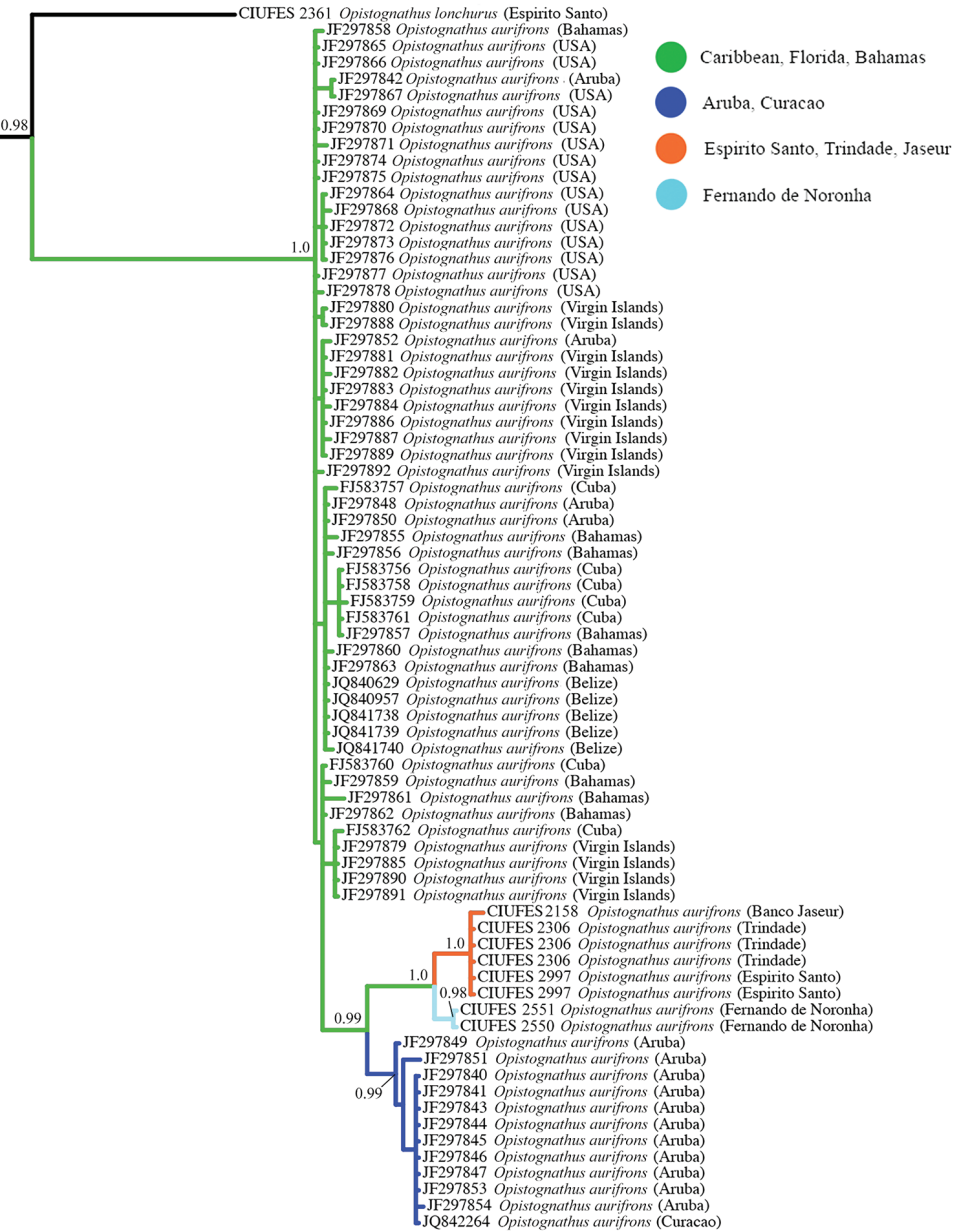
Bayesian inference phylogeny of the *Opistognathusaurifrons* complex. Support values are Bayesian posterior probabilities. Nodes without support values shown have <0.90 support. Scale bar units are expected number of substitutions per site.

**Figure 25.**
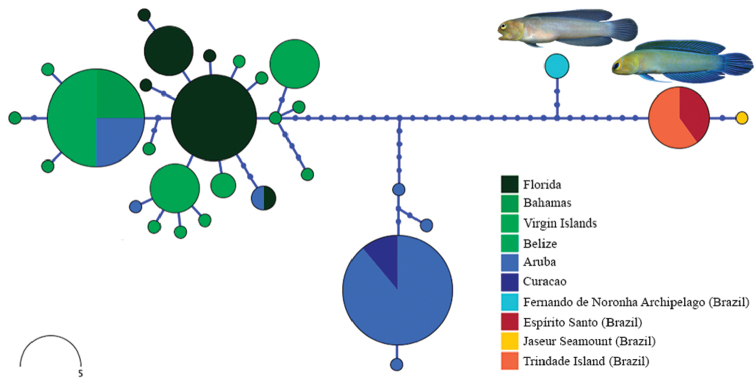
Haplotype network of the *Opistognathusaurifrons* complex. Dark solid circles represent un-sampled intermediate haplotypes, lines connecting haplotypes represent single substitutions.

Two groups in our initial analysis showed distinct phylogenetic structure and geographic genetic variation that suggested the presence of cryptic species. In the first case, specimens initially identified as *O.whitehursti* from Brazil and the Caribbean each formed two reciprocally monophyletic clades with considerable genetic differentiation in COI (mean between group p-distance = 0.11 (mean within group p-distance ≤ 0.003, Table 2). Subsequent analysis of coloration and morphology revealed subtle differences that support the recognition of these mitochondrial lineages as distinct species (see “Comparisons” in *O.vicinus* description). *Opistognathusaurifrons* formed four distinct groups (Figs 24–25), with one large polyphyletic grade from the Caribbean, a clade nested with this grade from Aruba and Curacao, a clade from mainland Brazil and Vitória-Trindade Seamounts Chain, and a pair of sequences from Fernando de Noronha Archipelago. Genetic variation in COI was very low within each of these groups (mean p-distance ≤0.006), but moderate between each group (mean p-distance between groups = 0.016–0.045). A haplotype map of the *Opistognathusaurifrons* complex (Figure 25) confirms the four main clusters of haplotypes, which are separated from one another by at least nine mutations in the partial COI gene. The strong connection between the Trindade and mainland populations is probably related to the stepping-stone process provided by Vitória-Trindade Seamounts Chain (Floeter and Gasparini 2000; Macieira et al. 2015; Pinheiro et al. 2017, 2018).

Previous studies of several populations of Caribbean *O.aurifrons* based on morphology (Böhlke and Thomas 1961) and genetics (Ho et al. 2012) indicated that despite substantial variation in color pattern within and between populations, only a single species should be recognized. Several other western Atlantic reef fishes, especially small, cryptobenthic fishes like gobies and blennioids, show similar patterns where there is substantial mitochondrial divergence without detectable phenotypic differences (Victor et al. 2015; Victor 2015). Some examples include the multiple genetic lineages within *Bathygobiussoporator* (Tornabene et al. 2010; Tornabene and Pezold 2011), *Malactoctenustriangulatus* (Victor 2015), several species of *Starksia* (Baldwin et al. 2011), and some species in the *Tigrigobius (Elacatinus) multifasciatus/panamensis* complex (Victor 2010, 2014). In these instances, nominal species comprise multiple highly-divergent mitochondrial lineages, each with very little genetic variation within lineages, and no recognizable phenotypic differences between lineages (such groups have been termed “genovariants” by Victor 2015). In these studies, and in most cases of such ‘cryptic species’ of reef fishes, authors have conservatively refrained from naming them as new species based solely on the presence reciprocally monophyletic mitochondrial lineages. Part of this is for practical reasons; taxonomists often prefer species descriptions to be operational, and thus having observable diagnostic characters is critically important. However, it is also possible that such genetic lineages don’t correspond to distinct species, and instead are the result of incomplete lineage sorting, or in the case of allopatric lineages, simply representative of geographic population-genetic structure. For these reasons, we follow convention and refrain from naming the distinct lineages of *O.aurifrons*, pending additional genetic and phenotypic data. However, for conservation purposes it is important to consider these four lineages separately.

## Supplementary Material

XML Treatment for Opistognathus thionyi

XML Treatment for Opistognathus vicinus

XML Treatment for Opistognathus brasiliensis

XML Treatment for Opistognathus cuvierii

XML Treatment for Opistognathus lonchurus

XML Treatment for Opistognathus aff. aurifrons

## Appendix

**Table:**

Genus	species	GenBank Accession Number	Voucher from this study
* Lonchopisthus *	* micrognathus *	MH751526	USNM 438666
* Opistognathus *	* afer *	KU176393	n/a
* Opistognathus *	* aurifrons *	JQ842264	n/a
* Opistognathus *	* aurifrons *	JQ841740	n/a
* Opistognathus *	* aurifrons *	JQ841739	n/a
* Opistognathus *	* aurifrons *	JQ841738	n/a
* Opistognathus *	* aurifrons *	JQ840957	n/a
* Opistognathus *	* aurifrons *	JQ840629	n/a
* Opistognathus *	* aurifrons *	JF297892	n/a
* Opistognathus *	* aurifrons *	JF297891	n/a
* Opistognathus *	* aurifrons *	JF297890	n/a
* Opistognathus *	* aurifrons *	JF297889	n/a
* Opistognathus *	* aurifrons *	JF297888	n/a
* Opistognathus *	* aurifrons *	JF297887	n/a
* Opistognathus *	* aurifrons *	JF297886	n/a
* Opistognathus *	* aurifrons *	JF297885	n/a
* Opistognathus *	* aurifrons *	JF297884	n/a
* Opistognathus *	* aurifrons *	JF297883	n/a
* Opistognathus *	* aurifrons *	JF297882	n/a
* Opistognathus *	* aurifrons *	JF297881	n/a
* Opistognathus *	* aurifrons *	JF297880	n/a
* Opistognathus *	* aurifrons *	JF297879	n/a
* Opistognathus *	* aurifrons *	JF297878	n/a
* Opistognathus *	* aurifrons *	JF297877	n/a
* Opistognathus *	* aurifrons *	JF297876	n/a
* Opistognathus *	* aurifrons *	JF297875	n/a
* Opistognathus *	* aurifrons *	JF297874	n/a
* Opistognathus *	* aurifrons *	JF297873	n/a
* Opistognathus *	* aurifrons *	JF297872	n/a
* Opistognathus *	* aurifrons *	JF297871	n/a
* Opistognathus *	* aurifrons *	JF297870	n/a
* Opistognathus *	* aurifrons *	JF297869	n/a
* Opistognathus *	* aurifrons *	JF297868	n/a
* Opistognathus *	* aurifrons *	JF297867	n/a
* Opistognathus *	* aurifrons *	JF297866	n/a
* Opistognathus *	* aurifrons *	JF297865	n/a
* Opistognathus *	* aurifrons *	JF297864	n/a
* Opistognathus *	* aurifrons *	JF297863	n/a
* Opistognathus *	* aurifrons *	JF297862	n/a
* Opistognathus *	* aurifrons *	JF297861	n/a
* Opistognathus *	* aurifrons *	JF297860	n/a
* Opistognathus *	* aurifrons *	JF297859	n/a
* Opistognathus *	* aurifrons *	JF297858	n/a
* Opistognathus *	* aurifrons *	JF297857	n/a
* Opistognathus *	* aurifrons *	JF297856	n/a
* Opistognathus *	* aurifrons *	JF297855	n/a
* Opistognathus *	* aurifrons *	JF297851	n/a
* Opistognathus *	* aurifrons *	JF297854	n/a
* Opistognathus *	* aurifrons *	JF297853	n/a
* Opistognathus *	* aurifrons *	JF297852	n/a
* Opistognathus *	* aurifrons *	JF297850	n/a
* Opistognathus *	* aurifrons *	JF297849	n/a
* Opistognathus *	* aurifrons *	JF297848	n/a
* Opistognathus *	* aurifrons *	JF297847	n/a
* Opistognathus *	* aurifrons *	JF297846	n/a
* Opistognathus *	* aurifrons *	JF297845	n/a
* Opistognathus *	* aurifrons *	JF297844	n/a
* Opistognathus *	* aurifrons *	JF297843	n/a
* Opistognathus *	* aurifrons *	JF297842	n/a
* Opistognathus *	* aurifrons *	JF297841	n/a
* Opistognathus *	* aurifrons *	JF297840	n/a
* Opistognathus *	* aurifrons *	FJ583762	n/a
* Opistognathus *	* aurifrons *	FJ583761	n/a
* Opistognathus *	* aurifrons *	FJ583760	n/a
* Opistognathus *	* aurifrons *	FJ583759	n/a
* Opistognathus *	* aurifrons *	FJ583758	n/a
* Opistognathus *	* aurifrons *	FJ583757	n/a
* Opistognathus *	* aurifrons *	FJ583756	n/a
* Opistognathus *	aff. aurifrons	MH751545	CIUFES 2158
* Opistognathus *	aff. aurifrons	MH751543	CIUFES 2306
* Opistognathus *	aff. aurifrons	MH751544	CIUFES 2306
* Opistognathus *	aff. aurifrons	MH751542	CIUFES 2306
* Opistognathus *	aff. aurifrons	MH751538	CIUFES 2550
* Opistognathus *	aff. aurifrons	MH751539	CIUFES 2551
* Opistognathus *	aff. aurifrons	MH751541	CIUFES 2997
* Opistognathus *	aff. aurifrons	MH751540	CIUFES 2997
* Opistognathus *	* aff. jacksoniensis *	JF911713	n/a
* Opistognathus *	* lonchurus *	MH751546	CIUFES 2361
* Opistognathus *	* macrognathus *	JQ841946	n/a
* Opistognathus *	* macrognathus *	JN193388	n/a
* Opistognathus *	* maxillosus *	JQ840631	n/a
* Opistognathus *	* maxillosus *	JQ840630	n/a
* Opistognathus *	* muscatensis *	KU176461	n/a
* Opistognathus *	* muscatensis *	KU176379	n/a
* Opistognathus *	* robinsi *	JN193389	n/a
* Opistognathus *	* thionyi *	MH751535	CIUFES 2054
* Opistognathus *	* thionyi *	MH751530	CIUFES 2341
* Opistognathus *	* thionyi *	MH751531	CIUFES 2341
* Opistognathus *	* thionyi *	MH751532	CIUFES 2341
* Opistognathus *	* thionyi *	MH751536	CIUFES 2394
* Opistognathus *	* thionyi *	MH751537	CIUFES 2394
* Opistognathus *	* thionyi *	MH751534	CIUFES 2421
* Opistognathus *	* thionyi *	MH751533	CIUFES 2426
* Opistognathus *	* vicinus *	MH751528	CIUFES 0236
* Opistognathus *	* vicinus *	MH751527	CIUFES 2362
* Opistognathus *	* vicinus *	MH751529	CIUFES 2395
* Opistognathus *	* brasiliensis *	MH751525	CIUFES 3361
* Opistognathus *	* maxillosus *	LIDM1299-8*	n/a
* Opistognathus *	* whitehursti *	LIDMA332-10*	n/a
* Opistognathus *	* whitehursti *	LIDMA349-10*	n/a
* Opistognathus *	* whitehursti *	MFLII323-7*	n/a
* Opistognathus *	* whitehursti *	MFLII324-7*	n/a
* Opistognathus *	* whitehursti *	MH751547	UF 183104

*Numbers from Barcode of Life Databse (BOLD), not GenBank.

## References

[B1] Baldwin CC Castillo C Weigt LA Victor BC (2011) Seven new species within western Atlantic *Starksiaatlantica*, *S.lepicoelia*, and *S.sluiteri* (Teleostei, Labrisomidae), with comments on the congruence of DNA barcodes and species. ZooKeys 79: 21–72. 10.3897/zookeys.79.1045 PMC3088046 21594143

[B2] Böhlke JE Chaplin CCG (1968) Fishes of the Bahamas and adjacent waters. 2^nd^ ed. University of Texas Press, Austin, 769 pp.

[B3] Böhlke JE Thomas LP (1961) Notes on the west Atlantic jawfishes, *Opisthognathusaurifrons*, *O.lonchurus* and *Gnathypopsbermudezi*. Bulletin of Marine Science of the Gulf and Caribbean 11(4): 503–516.

[B4] Brasil (2018) Decreto N° 9.312, de 19 de março de 2018. DOU, Brasília, BR, 1–3.

[B5] Briggs JC Bowen BW (2012) A realignment of marine biogeographic providences with particular references to fish distributions. Journal of Biography 39: 12–30.

[B6] Bussing WA Lavenberg RJ (2003) Four new species of eastern tropical Pacific jawfishes (*Opistognathus*: Opistognathidae). Revista de Biologia Tropical 51(2): 529–550. 15162745

[B7] Carvalho-Filho A (1999) Peixes: costa brasileira. Melro, São Paulo, 320 pp.

[B8] Colin PL (1971) Interspecific relationships of the Yellowhead Jawfish, *Opistognathusaurifrons* (Pisces, Opistognathidae). Copeia 1971(3): 469–473. 10.2307/1442443

[B9] Colin PL (1973) Copeia 1973(1): 84–90. 10.2307/1442361

[B10] Cuvier G (1816) *Le regne animal*. 1st ed., Paris 2, 532 pp.

[B11] Cuvier G (1836) Histoire naturelle des poissons. Tome onzième. Livre treizième. De la famille des Mugiloïdes. Livre quatorzième. De la famille des Gobioïdes 11: 506 pp.

[B12] Feitoza BM Rosa RS Rocha LA (2005) Ecology and zoogeography of deep-reef fishes in northeastern Brazil. Bulletin of Marine Science 76(3): 725–742.

[B13] Floeter SR Gasparini JL (1999) Ocorrȇncia de *Opistognathuslonchurus* Jordan & Gilbert, 1882 (Perciformes: Opistognathidae) na costa Brasileira. Resumos XII Encontro Brasileiro de Ictiologia 56: 58.

[B14] Fricke R Eschmeyer WN (2018) Guide to fish collections. http://researcharchive.calacademy.org/research/ichthyology/catalog/collections.asp) California Academy of Sciences, CA.

[B15] Floeter S Gasparini JL (2000) The southwestern Atlantic reef fish fauna: composition and zoogeographic patterns. Journal of Fish Biology 56: 1099–1114. 10.1111/j.1095-8649.2000.tb02126.x

[B16] Gasparini JL Floeter SR (2001) The shore fishes of Trindade Island, western South Atlantic. Journal of Natural History 35(11): 1639–1656.

[B17] Giglio VJ Pinheiro HT Bender MG Bonaldo RM Costa-Lotufo LV Ferreira CEL Floeter SR Freire A Gasparini JL Joyeux J-C Krajewski JP Lindner A Longo GO Lotufo TMC Loyola R Luiz OJ Macieira RM Magris RA Mello TJ Quimbayo JP Rocha LA Segal B Teixeira JB Vila-Nova DA Vilar CC Zilberberg C Francini-Filho RB (2018) Large and remote marine protected areas in the South Atlantic Ocean are flawed and raise concerns: Comments on Soares and Lucas (2018). Marine Policy 96: 13–17. 10.1016/j.marpol.2018.07.017

[B18] Hess HC (1993) Male mouthbrooding in jawfishes (Opistognathidae): constraints on polygyny. Bulletin of Marine Science 52: 806–818.

[B19] Ho ALFC Pruett CL Lin J (2012) Population genetic structure, coloration, and morphometrics of yellowhead jawfish *Opistognathusaurifrons* (Perciformes: Opistognathidae) in the Caribbean region. Marine Ecology Progress Series 444: 275–287. 10.3354/meps09435

[B20] Jordan DS Thompson JC (1905) The fish fauna of the Tortugas Archipelago. Bulletin of the Bureau of Fisheries 24 (for 1904): 229–256.

[B21] Jordan DS Gilbert CH (1882) Notes on fishes observed about Pensacola, Florida, Florida, and Galveston, Texas, with description of new species. Proceedings of the United States National Museum 5(282): 241–307. 10.5479/si.00963801.5-282.241

[B22] Lanfear R Frandsen PB Wright AM Senfeld T Calcott B (2016) PartitionFinder 2: new methods for selecting partitioned models of evolution for molecular and morphological phylogenetic analyses. Molecular Biology and Evolution 34(3): 772–773. 10.1093/molbev/msw260 28013191

[B23] Longley WH (1927) Observations upon the ecology of Tortugas fishes with notes upon the taxonomy of species new or little known. Carnegie Institution of Washington year book 26: 222–224.

[B24] Longley WH Hildebrand SF (1941) Systematic catalogue of the fishes of Tortugas, Florida. Carnegie Institution of Washington publication 535: 225–285.

[B25] Macieira RM Simon T Pimentel CR Joyeux J-C (2015) Isolation and speciation of tidepool fishes as a consequence of Quaternay sea-level fluctuations. Environmental Biology of Fishes 98: 385–393. 10.1007/s10641-014-0269-0

[B26] Magris RA Pressey RL (2018) Marine protected areas; Just for show? Science 360: 723–724. 10.1126/science.aat6215 29773739

[B27] Menezes NA (2011) Checklist dos peixes marinhos do Estado de São Paulo, Brasil. Biota Neotropica 11: 33–46. 10.1590/S1676-06032011000500003

[B28] Menezes NA Buckup PA Figueiredo JL Moura RL (2003) Catálogo das espécies de peixes marinhos do Brasil. Museu de Zoologia da Universidade de São Paulo, São Paulo, 160 pp.

[B29] Menezes NA Figueiredo JL (1985) Manual de peixes marinhos do sudeste do Brasil - Volume V (Teleostei 4). Museu de Zoologia, Universidade de São Paulo, São Paulo, 105 pp.

[B30] Mincarone MM Martins AS Costa PASd Braga AdC Haimovici M (2017) Peixes marinhos da Bacia de Campos: uma revisão da diversidade. In: Curbelo-Fernandez MP Braga AC (Eds) Comunidades Demersais e Bioconstrutores: caracterização ambiental regional da Bacia de Campos, Atlântico Sudoeste.Elsevier, Rio de Janeiro, 187–216. 10.1016/B978-85-352-7295-6.50008-7

[B31] Moura de RL Gasparini JL Sazima I (1999) New records and range extensions of reef fishes in the western south Atlantic, with comments on reef fish distribution along the Brazilian coast. Revista Brasileira de Zoologia 16(2): 513–530. 10.1590/S0101-81751999000200017

[B32] Moura RL Sazima I (2000) Species richness and endemism levels of the Southwestern Atlantic reef fish fauna. In: Moosa MK Soemodihardjo S Soegiarto A Romimohtarto K Nontji A Soekarno Suharsono (Eds) Proceedings of the 9th International Coral Reef Symposium.Ministry of Environment, the Indonesian Institute of Sciences and the International Society for Reef Studies, Bali, Indonesia, 481–486.

[B33] Page LM Espinosa-Pérez H Findley LT Gilbert CR Lea RN Mandrak NE Mayden RL Nelson JS (2013) Common and scientific names of fishes from the United States, Canada, and Mexico, 7^th^ edition. American Fisheries Society Special Publication 34, 384 pp.

[B34] Pereira-Filho GH Veras PC Francini-Filho RB Moura RL Pinheiro HT Gibran FZ Matheus Z Neves LM Amado-Filho GM (2015) Effects of the sand tilefish *Malacanthusplumieri* on the structure and dynamics of a rhodolith bed in the Fernando de Noronha Archipelago, tropical West Atlantic. Marine Ecology Progress Series 541: 65–73. 10.3354/meps11569

[B35] Pinheiro HT Bernardi G Simon T Joyeux J-C Macieira RM Gasparini JL Rocha C Rocha LA (2017) Island biogeography of marine organisms. Nature 549: 82–85. 10.1038/nature23680 28854164

[B36] Pinheiro HT Mazzei E Moura RL Amado-Filho GM Carvalho-Filho A Braga AC Costa PAS Ferreira BP Ferreira CEL Floeter SR Francini-Filho RB Gasparini JL Macieira RM Martins AS Olavo G Pimentel CR Rocha LA Sazima I Simon T Teixeira JB Xavier LB Joyeux J-C (2015) Fish biodiversity of the Vitória-Trindade Seamount Chain, southwestern Atlantic: An updated database. PLoS ONE 10: e0118180 10.1371/journal.pone.0118180 PMC4349783 25738798

[B37] Pinheiro HT Rocha LA Macieira RM Carvalho-Filho A Anderson AB Bender MG Di Dario F Ferreira CEL Figueiredo-Filho J Francini-Filho R Gasparini JL Joyeux J-C Luiz OJ Mincarone MM Moura RL Nunes JdACC Quimbayo JP Rosa RS Sampaio CLS Sazima I Simon T Vila-Nova DA Floeter SR (2018) South-western Atlantic reef fishes: Zoogeographical patterns and ecological drivers reveal a secondary biodiversity centre in the Atlantic Ocean. Diversity and Distributions: 24: 951–965. 10.1111/ddi.12729

[B38] Pinto SY (1970) Observações ictiologicas. III Sobre *Opistognathuscuvieri* Valenciennes, 1836 (Actinopterygi, Perciformes, Opisthognathidae). Atas da Sociedade de Biologia do Rio de Janeiro 13: 3–4.

[B39] Poey F (1860) Poissons de Cuba In: Memorias sobre la historia natural de la isla de Cuba, acompanados de sumarios Latinos y extractos en Francés 2: 115–356.

[B40] Robertson DR Van Tassell J (2015) Shorefishes of the Greater Caribbean: online information system. Version 1.0, Smithsonian Tropical Research Institute, Balboa, Panamá. Electronic version accessed 4 October 2017 at http://biogeodb.stri.si.edu/caribbean/en/pages

[B41] Rocha LA (2002) Brazilian reef fishes. In: P. Humann and N. Deloach, eds. Reef fish identification - Florida, Caribbean, Bahamas, 3^rd^ ed. New World Publications, Jacksonville, 462–479.

[B42] Rocha LA Rosa LL (2001) Baseline assessment of reef fish assemblages of Parcel Manuel Luiz Marine State Park, Maranhāo, north-east Brazil. Journal of Fish Biology 58(4): 985–998.

[B43] Ronquist F Meslenko M van der Mark P Ayres DL Darling A Höhna S Larget B Liu L Suchard MA Huelsenbeck JP (2012) Mr Bayes 3.2: efficient Bayesian phylogenetic inference and model choice across a large model space. Systematic Biology 61(3): 539–542. 22357727 10.1093/sysbio/sys029 PMC3329765

[B44] Roux C (1964) Les Côtes du Brésil et l’Histoire Naturelle des Poissons de Cuvier et Valenciennes. Mélanges ichthyologiques. Mémoires I.F.A.N. 68: 385–435.

[B45] Roux C (1973) Poissons teleosteens du plateau continental bresilien, resultats scientifiques des campagnes dela “Calypso” X. Annales l’Institut Océanographique, Monaco (Nouvelle Série) 49, fasc. Supplementaire: 23–207.

[B46] Salzburger W Ewing GB Von Haeseler A (2011) The performance of phylogenetic algorithms in estimating haplotype genealogies with migration. Molecular Ecology 20: 1952–1963. 21457168 10.1111/j.1365-294X.2011.05066.x

[B47] Sampaio CLS Nottingham MC (2008) Guia para identificação de peixes ornamentais: espécies marinhas. Ibama, Brasília, 205 pp.

[B48] Shubart CD Santl T Koller P (2007) Mitochondrial patterns of intra- and interspecific differentiation among endemic freshwater crabs of ancient lakes in Sulawesi. Contributions to Zoology 77(2): 93–90.

[B49] Simon T Macieira RM Joyeux J-C (2013a) The shore fishes of the Trindade-Martin Vaz insular complex: an update. Journal of Fish Biology 82: 2113–2127. 23731156 10.1111/jfb.12126

[B50] Simon T Joyeux J-C Pinheiro HT (2013b) Fish assemblages on shipwrecks and natural rocky reefs strongly differ in trophic structure. Marine Environmental Research 90: 55–65. 23796542 10.1016/j.marenvres.2013.05.012

[B51] Smith-Vaniz WF (1997) Descriptions of five new species of jawfishes (*Opistognathus*: Opistognathidae) from the western Atlantic Ocean. Bulletin of Marine Science 60(3): 1074–1128.

[B52] Smith-Vaniz WF (2017) Descriptions of a new genus and two new species of Caribbean deep-water jawfishes (Teleostei: Opistognathidae). Journal of the Ocean Science Foundation 26: 46–58.

[B53] Smith-Vaniz WF Jelks HL (2014) Marine and inland fishes of St. Croix, U. S. Virgin Islands; an annotated checklist. Zootaxa monograph 3803: 1–120. 10.11646/zootaxa.3803.1.1 24871150

[B54] Smith-Vaniz WF Williams JT Curtis M Pina Amargos F Brown J (2015) *Opistognathuslonchurus* The IUCN Red List of Threatened Species 2015: e.T16546023A16546264. 10.2305/IUCN.UK.2015-2.RLTS.T16546023A16546264.en

[B55] Smith-Vaniz WF Walsh SJ (2017) Revision of the jawfish genus *Lonchopisthus* with description of a new Atlantic species (Teleostei: Opistognathidae). Journal of the Ocean Science Foundation 28: 52–89.

[B56] Stamatakis A (2014) RAxML Version 8: a tool for phylogenetic analysis and post-analysis of large phylogenies. Bioinformatics 30(9): 1312–1313. 10.1093/bioinformatics/btu033 24451623 PMC3998144

[B57] Stocco LB Joyeux J-C (2015) Distribution of fish larvae on the Vitória-Trindade Chain, southwestern Atlantic. Check List 11: 1590. 10.15560/11.2.1590

[B58] Thacker CE (2003) Molecular phylogeny of the gobioid fishes. Molecular Phylogenetics and Evolution 26(3): 354–368. 10.1016/S1055-7903(02)00361-5 12644397

[B59] Tornabene L Baldwin C Weigt LA Pezold F (2010) Exploring the diversity of western Atlantic *Bathygobius* (Teleostei: Gobiidae) with cytochrome x oxidase-I, with descriptions of two new species. Aqua, International Journal of Ichthyology 16(4): 141–170.

[B60] Tornabene L Pezold F (2011) Phylogenetic analysis of Western Atlantic *Bathygobius* (Teleostei: Gobiidae). Zootaxa 3042: 27–36.

[B61] Victor BC (2010) The Redcheek Paradox: the mismatch between genetic and phenotypic divergence among deeply divided mtDNA lineages in a coral-reef goby, with the description of two new cryptic species from the Caribbean Sea. Journal of the Ocean Science Foundation 3: 2–16.

[B62] Victor BC (2014) Three new endemic cryptic species revealed by DNA barcoding of the gobies of the Cayman Islands (Teleostei: Gobiidae). Journal of the Ocean Science Foundation 12: 25–60.

[B63] Victor BC (2015) How many coral reef fish species are there? Cryptic diversity and the new molecular taxonomy. In: Mora C (Ed.) Ecology of Fishes on Coral Reefs.Cambridge University Press, Cambridge, 76–88.

[B64] Victor BC Valdez-Moreno M Vásquez-Yeomans L (2015) Status of DNA Barcoding Coverage for the Tropical Western Atlantic Shorefishes and Reef Fishes. DNA Barcodes 3: 85–93. 10.1515/dna-2015-0011

